# Design and preliminary verification of a novel powered ankle–foot prosthesis: From the perspective of lower-limb biomechanics compared with ESAR foot

**DOI:** 10.1371/journal.pone.0303397

**Published:** 2024-06-07

**Authors:** Jingjing Liu, Jingang Liu, Pei Yi Cheah, Mouaz Al Kouzbary, Hamza Al Kouzbary, Selina X. Yao, Hanie Nadia Shasmin, Nooranida Arifin, Nasrul Anuar Abd Razak, Noor Azuan Abu Osman

**Affiliations:** 1 Centre for Applied Biomechanics, Department of Biomedical Engineering, Faculty of Engineering, Universiti Malaya, Kuala Lumpur, Malaysia; 2 School of Mechanical Engineering and Mechanics, Xiangtan University, Xiangtan, Hunan, China; 3 Centre for Sports and Exercise Science, Universiti Malaya, Kuala Lumpur, Malaysia; 4 Department of Mechanical Engineering, University of Vermont, Burlington, Vermont, United States of America; 5 The Chancellery, Universiti Tenaga Nasional, Kajang, Malaysia; Polytechnic University of Marche: Universita Politecnica delle Marche, ITALY

## Abstract

A novel powered ankle–foot prosthesis is designed. The effect of wearing the novel prosthesis and an energy-storage-and-return (ESAR) foot on lower-limb biomechanics is investigated to preliminarily evaluate the design. With necessary auxiliary materials, a non-amputated subject (a rookie at using prostheses) is recruited to walk on level ground with an ESAR and the novel powered prostheses separately. The results of the stride characteristics, the ground reaction force (GRF) components, kinematics, and kinetics in the sagittal plane are compared. Wearing the powered prosthesis has less prolongation of the gait cycle on the unaffected side than wearing the ESAR foot. Wearing ESAR or proposed powered prostheses influences the GRF, kinematics, and kinetics on the affected and unaffected sides to some extent. Thereinto, the knee moment on the affected side is influenced most. Regarding normal walking as the reference, among the total of 15 indexes, the influences of wearing the proposed powered prosthesis on six indexes on the affected side (ankle’s/knee’s/hip’s angles, hip’s moment, and Z- and X-axis GRF components) and five indexes on the unaffected side (ankle’s/knee’s/hip’s angles and ankle’s/hip’s moments) are slighter than those of wearing the ESAR foot. The influences of wearing the powered prosthesis on two indexes on the unaffected side (knee’s moment and X-axis GRF component) are similar to those of wearing the ESAR foot. The greatest improvement of wearing the powered prosthesis is to provide further plantarflexion after reaching the origin of the ankle joint before toe-off, which means that the designed powered device can provide further propulsive power for the lifting of the human body’s centre of gravity during walking on level ground. The results demonstrate that wearing the novel powered ankle–foot prosthesis benefits the rookie in recovering the normal gait more than wearing the ESAR foot.

## Introduction

Transtibial amputation is one of the most frequently conducted amputation surgeries in the world, which leads to amputees’ loss of locomotion ability. Reestablishment of independent and functional walking is one of the main focuses of rehabilitation for lower-limb amputees [1], which relies on the utilisation of the prostheses for transtibial amputees (also known as ankle–foot prostheses or below-knee prostheses). A complete system of the ankle–foot prosthesis worn by transtibial amputees usually includes the liner with the suspension, socket, pylon, and prosthesis. Although each component and the alignment between components have an influence on the comfort and rehabilitation degree of users [2–6], the prosthesis is the most important part of recovering the partial function of the sound muscles and ankle joint.

Different types of ankle–foot prostheses have been developed. Solid-ankle cushion-heel (SACH) foot is still one of the most used prostheses after about 70 years of introducing its standard design [7] because of its low price. However, since the SACH foot has no adaption for different terrains, amputees with moderate to high activity prefer a more advanced kind of prostheses––energy storage and return (ESAR) prostheses. The largest two suppliers of prostheses in the world provide tens of designs of ESAR, such as Pro-Flex® XC from Össur [8], Iceland and Restore from Ottobock [9]. ESAR feet are constructed of elastic materials (usually carbon or glass fibre), which deform under load and store elastic potential energy in the early stage of the stance phase and release in the late stage of the stance phase when the elastic materials return to their equilibrium position [10]. However, an obvious defect of ESAR feet is that they cannot provide net positive power, unlike the sound ankle joint. To remedy the defect, researchers paid attention to the powered ankle–foot prosthesis as a new direction of development. Since the powered prosthesis was first proposed in 1998 [11], more than 90 different kinds of designs were reported according to our previous statistics [12]. In this case, it is worth studying whether wearing the powered ankle–foot prosthesis can provide a better rehabilitation extent than wearing the ESAR foot for the users as expected.

Including the direct comparison between the performance of different prostheses and the sound ankle joint, investigating the influence of wearing different prostheses on other physiological indexes or sound lower-limb joints’ biomechanics is also essential to indirectly evaluate the devices. Some examples related to different transfemoral prostheses have been reported [13–15]. Regarding ankle–foot prostheses, combining the results of a systematic review [16] with our best knowledge, we find that the literature related to the above issue is limited. All these studies reviewed below [17–25] are based on the first commercialised powered ankle–foot prosthesis termed BiOM, of which the design and control were reported in [26–28]. The physiological indexes investigated in the literature are diverse, including metabolic costs [17–19], step-to-step transition work [17, 18], preferred walking speed [17, 19], and whole-body angular momentum [20]. Most results show that these indexes of wearing the powered ankle-foot prosthesis are closer to those of non-amputated data than the counterparts of wearing the ESAR foot. In addition, Russell Esposito et al. [18] also compared the joint’s displacement and moment outputted by different types of prostheses during level-ground walking and ascending slope. Compared with the control group, the ankle joint’s displacement when wearing the powered device is closer to that of the sound ankle joint than when wearing the ESAR foot, but the ankle joint’s moment is not. Grabowski and D’Andrea [21] reported the results of ground reaction forces (GRF) and knee joint kinetics of the unaffected leg at different walking speeds, which shows that wearing the powered device has a slight improvement with respect to wearing the ESAR foot.

Based on the same powered prosthesis BiOM, a series of studies were conducted with the same group of subjects [22–25]. The lower-limb joints’ angles, moments, and powers during level-ground walking, ascending slope, and ascending staircase are reported in [22–24], respectively. The results in [25] include the kinematics of lower-limb joints during walking on uneven ground. Among these results, except for the ankle joint’s angle outputted by different prostheses, the results of other lower-limb joints’ kinematics and kinetics from the groups of the powered device, ESAR foot, and non-amputated subjects do not show considerable differences. The above results are likely caused by the fact that the amputated subjects employed in these studies are highly active and trained military personnel who received months of intensive rehabilitation. Their physical coordination and adaptive capacity to different prostheses are much better than normal users, especially rookies unfamiliar with using ankle-foot prostheses. Therefore, for rookies, when they start to wear ankle-foot prostheses, it is very probable that the influence on their lower-limb biomechanics will not be as slight as the above situation. Consequently, it may cause some harm to the users’ musculoskeletal system. To our knowledge, no study related to the rookie has yet to be conducted.

With a rookie subject of using prostheses, this study conducted a preliminary verification of a novel design of powered ankle–foot prosthesis, which is based on a new elastic actuator. The lower-limb joints’ kinematics and kinetics of the subject during level-ground walking under different conditions are recorded and compared to evaluate the performance of the novel powered prosthesis. Simultaneously, this study also provides evidence to compare the effect of wearing the novel powered ankle–foot prosthesis and ESAR foot on lower-limb biomechanics for a rookie.

## Methods

### Design of a novel powered ankle-foot prosthesis

A novel powered ankle–foot prosthesis is designed and employed in this study. The novel prosthesis is based on a new elastic actuator—the unidirectional parallel elastic actuator with series elastic element (SE+UPEA), which has been compared with other elastic actuators in our previous study [29]. SE+UPEA is the best to optimise the speed and torque of the motor in the actuator [29]. That is, with the same motor, the powered ankle–foot prosthesis driven by the optimal SE+UPEA can provide more net power than other elastic actuators during the gait cycle.

**Fig 1:**
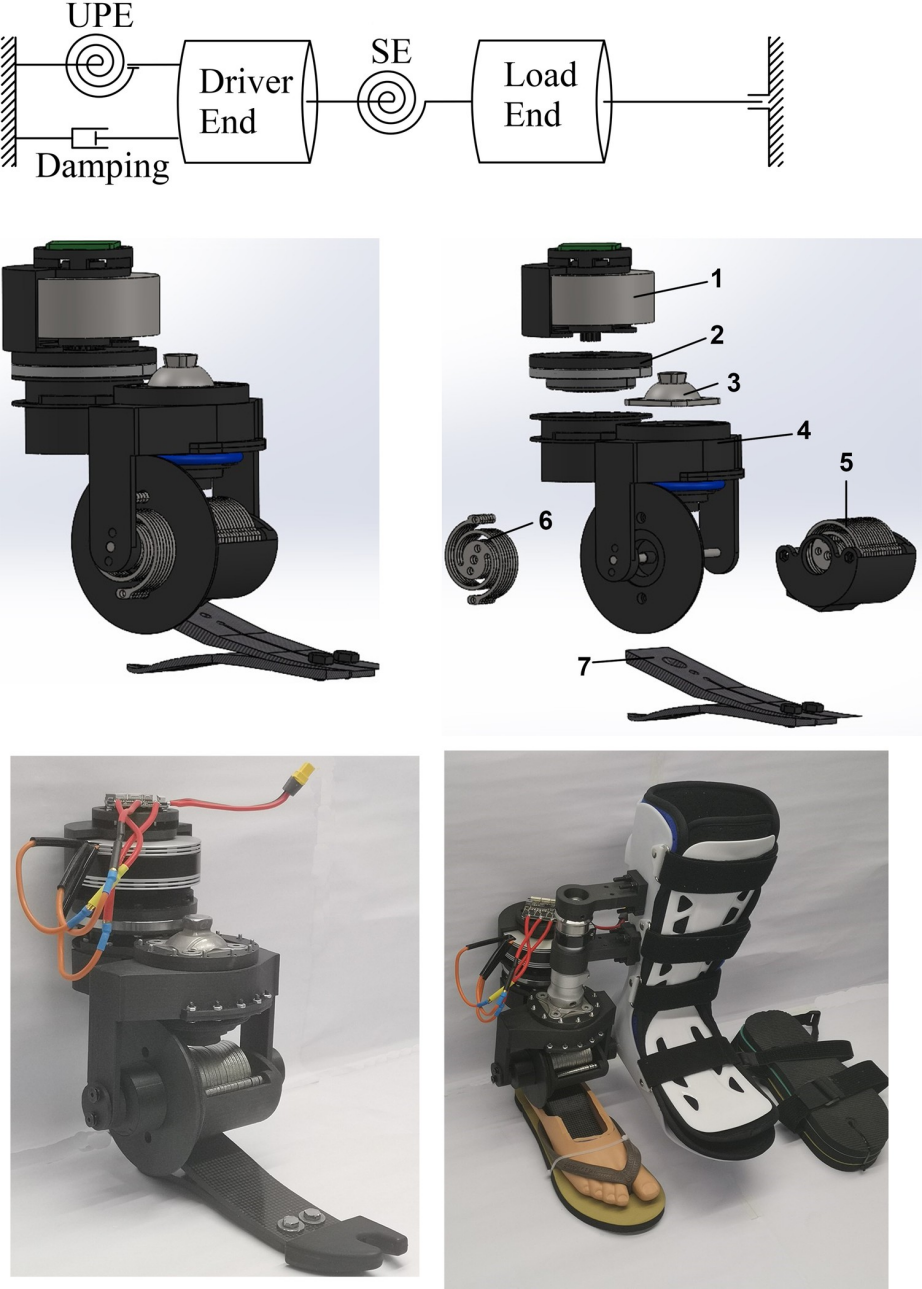
A novel powered ankle-foot prosthesis. (a) schematic diagram of SE+UPEA. (b) 3D model of the novel ankle-foot prosthesis. (c) exploded view of 3D model: 1–motor module, 2–harmonic drive module, 3–standard adapter, 4–two-stage cable drive module, 5–SE module, 6–UPE module, 7–ESAR foot part. (d) prototype used in the third and fourth trials. (e) prototype with the bypass system and auxiliary materials for the trials.

The schematic diagram of the SE+UPEA, as illustrated in Fig 1(A), consists of the driver end (the motor and transmission mechanisms), load end (except for the motor and transmission mechanisms, including all components of the prosthesis and all user’s body segments), damping on the driver end, and two sets of torsion springs. On the aspect of the damping, for the convenience of analysis, all the damping from the motor and transmission mechanisms, including the motor damping, the damping inside and between transmission components, and the damping between transmission components and the rack, is converted and unified equivalently to the damping between the driver end and the rack. Thereinto, the motor damping has the greatest influence on the system dynamics. Regarding torsion springs, the series elastic element (SE) is between the driver and load ends, and the unidirectional parallel elastic element (UPE) is between the driver end and the rack of the prosthesis. Take the stance phase of walking, except for the period from heel strike to foot flat, where the moment generated by the sound ankle joint follows the direction of plantarflexion to push off the body. Therefore, in order to mimic the sound ankle joint, the compression direction of the UPE is set as that of dorsiflexion so that it will generate the torque in the direction of plantarflexion when the UPE release the energy. In this case, the driver end can only compress the UPE along the dorsiflexion direction rather than extend it. The equilibrium position of the UPE can be set in the range of plantarflexion. In this case, the prosthesis provides a compressive preload of the UPE at its ankle joint origin to optimise the required output torque of the novel powered ankle–foot prosthesis’s driver unit. In order to ensure that the preload will not rotate the foot part, there are three available methods: a) the motor can be controlled to hold its initial position from the control perspective; b) an additional brake, such as an electromagnetic-actuated micro brake, can be employed to balance the preload during the non-operating period; c) some non-back-drivable transmission mechanisms can be used.

An optimisation method of elastic elements’ parameters, shown in the S1 File, has been reported in our previous study [29]. Briefly, in the optimisation method, given the specific ankle joint’s kinematics and kinetics as the output of the novel powered ankle–foot prosthesis, the required output performance of the driver end can be optimised by the parameters of SE and UPE based on the dynamic model of the SE+UPEA. Thereinto, the stiffness of SE is used to optimise the required output velocity of the driver end, whilst the parameters of UPE are used to optimise the required output torque of the driver end. For the subject recruited in this study, the optimal stiffness of the SE is 250.42 Nm/rad; the optimal stiffness and equilibrium position of the UPE are 92.89 Nm/rad and –0.37 rad, respectively. Based on the optimal parameters of SE and UPE, the required output power of SE+UPEA is minimised. It has been demonstrated in our previous study that the two-arm planar spiral spring based on the Archimedean spiral can be designed to provide enough stiffness and deformation range within the dimensional limitation [30].

It is worth noting that the stiffness of the foot part is not included in the abovementioned schematic diagram of the SE+UPEA. Since the load on the load end is in the format of torque, the calculation process has considered the deformation of the foot part, which is caused by the stiffness of the foot part. Therefore, the stiffness of the foot part is not included in the abovementioned schematic diagram of the SE+UPEA to avoid over-considering its influence.

Based on the SE+UPEA, following the conception of modular design, the mechanical structure of the novel powered ankle–foot prosthesis and its exploded view are illustrated in Fig 1(B) and 1(C). Except for the standard adapter and the foot part modified from a commercialised ESAR prosthesis, the mechanical design can be divided into five modules––the motor module, harmonic drive module, cable drive module, SE module, and UPE module. According to the ankle joint’s velocity and the torque load on the ankle joint of the subject recruited in this study during normal walking, of which the absolute maximums are about 4 rad/s and 1 Nm/kg, respectively, the following driver and transmission components are selected to have enough driving performance without considering the optimisation from elastic elements to guarantee the safety of the novel prosthesis. The motor is an outer-rotor brushless direct-current (DC) motor, MN801S KV150 (T-Motor, China). According to the official datasheet, under about 48 V, the selected motor can provide up to 3.75 Nm and 5247 rpm. A short and compact harmonic drive only with main gear components, CSD-25-120-2A-GR (Harmonic Drive SE, Germany), is selected. The reduction ratio of the harmonic drive is 120:1 when the circular spline is fixed, the wave generator is the input, and the flex spline is the output. The limit for momentary peak torque and maximal input speed are 152 Nm and 7000 rpm, respectively. The two-stage cable drive is in the open-loop format. The first-stage cable drive is used to achieve a function of parallel-axis transmission with a reduction ratio of 1:1, which allows the motor and harmonic drive to be assembled behind the adaptor and the pylon. The second-stage cable drive is designed to mimic a pair of bevel gears to achieve the intersect-axis transmission with a reduction ratio of 1.5:1. The 6 mm Dyneema rope, which is woven in ultra-high molecular weight polyethene, is used in the cable drive. The 6 mm Dyneema rope possesses a breaking strength of more than 30 kN with almost zero elongation rate, and it has a high resistance to bending fatigue and corrosion. The control board is an open-source design known as the DGM driver board, whose maximum continuous current is 30 A. Based on the introduced step-like structure, the built height of the prosthesis, which is from the top of the adaptor to the heel of the ESAR foot part, is about 175 mm. The weight of the prototype without batteries is 1901.3 g.

### Trials protocol

One non-amputated subject, who had never been trained to use a prosthesis, is recruited to walk on level ground, which is recorded by the optical motion capture system and force plate to obtain the kinetic and kinematic data. The recruited subject is a 31-year-old female whose height and body weight are 1.75 m and 64.5 Kg, respectively. She did not have any severe injury on the lower limb in the past year. The trials are approved by Universiti Malaya Research Ethics Committee (Reference No.: UM.TNC2/UMREC_2320). The recruited subject provided the informed consent in written form.

The walking trials of the subject include four separate trials, which are conducted on 2023/02/02, 2023/02/09, 2023/02/16, and 2023/02/23, respectively, and there is a one-week interval between each trial. For all the trials, the motion capture system with 12 cameras from Qualisys is set to 200 Hz to record the kinematic data, and the Gen 5 AMTI-NetForce (Watertown, MA, USA) with the size of 0.5 × 0.5*m*, which is set to 2000 Hz, is used. The kinetic data are obtained from the analysis of the software––Visual 3D (C-Motion, Inc, USA). During each trial, the subject walks forth and back on a level footpath, which is about 7 m in length and 1.5 m in width, for several minutes. The subject always wears shoes in all trials. As the whole footpath is covered, the subject does not know the position of the force plate. Therefore, it is believed that the subject did not deliberately change the stride to step on the force plate during the trials. In this study, only the GRF, kinematic, and kinetic results in the sagittal plane of these four trials are compared and discussed since the proposed powered ankle–foot prosthesis is designed to mimic the one-DOF motion in this plane.

**Fig 2:**
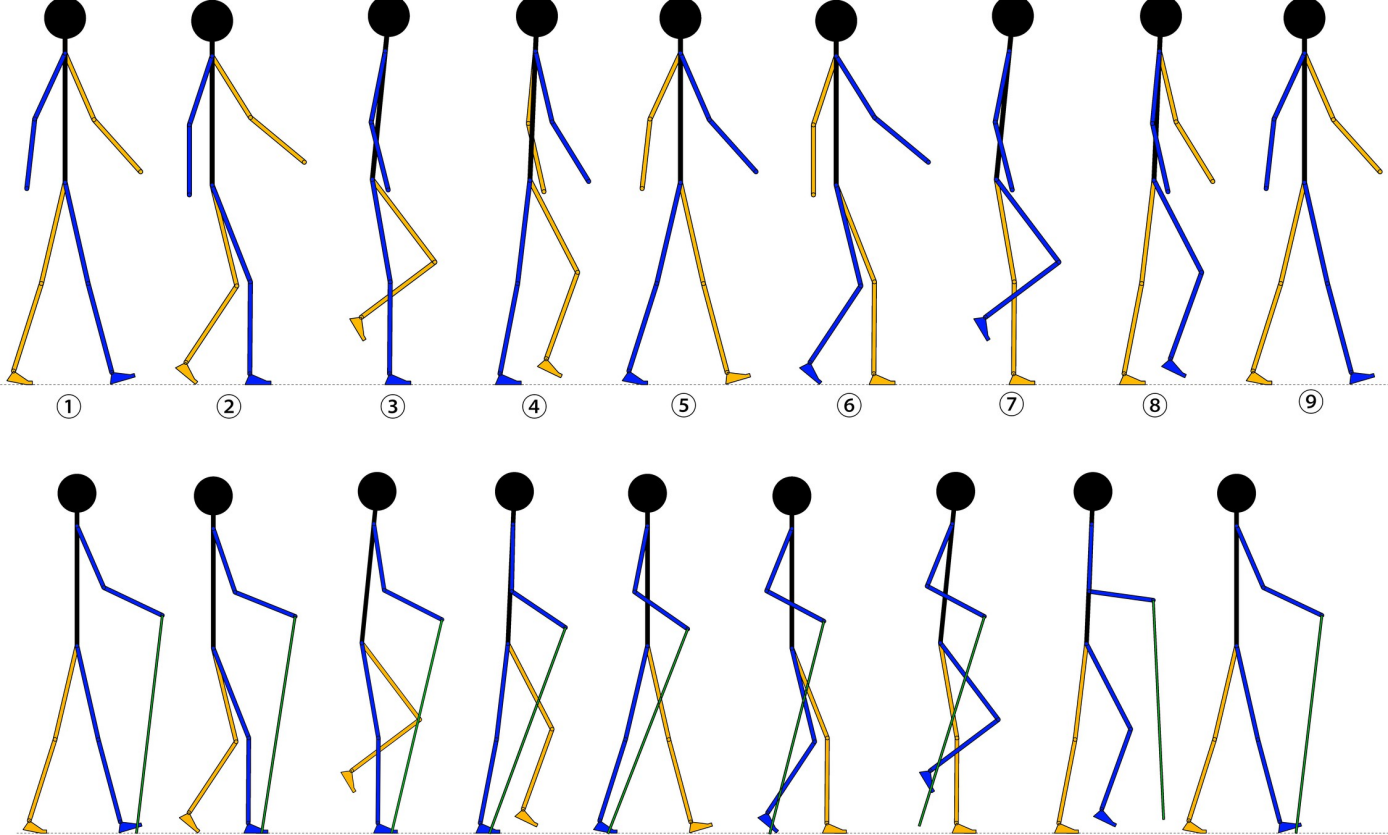
Diagram of typical motion in one gait cycle of normal walking and normal walking with sticks. (a) normal walking; (b) normal walking with sticks.

In the first trial, the subject is asked to complete the normal walking (NW) at a self-selected slow speed. The diagram of typical motion in one gait cycle of NW is shown in Fig 2(A). An entire gait cycle starts from the heel strike and ends with the next heel strike on the same side (from ① to ⑨). Thereinto, the phase from the first heel strike to the toe-off (from ① to ⑥) is the stance phase, and that from ⑥ to ⑨ is the swing phase. If the blue segments are on the right, the phase from the right heel strike (①) to the left toe-off (②) is the left double support phase, and that from the left heel strike (⑤) to the right toe-off (⑥) is the right double support phase. For the results of the first trial, the angular displacement of the right ankle joint will be set as the control target for the fourth trial. The GRF, kinematic, and kinetic results of other lower-limb joints are references to analyse the influence of wearing different prostheses.

Considering this study is the preliminary verification of the mechanical design scheme of the proposed powered ankle-foot prosthesis, the prototype worn by the recruited subject is prepared using the following steps. The most important task of this study is to verify the performance of the selected motor and transmission mechanism. Although the optimal parameters of the elastic elements have been obtained, the employment of elastic elements will improve the actuator’s performance by reducing the speed and torque requirement [29]. Therefore, on the aspect of mechanical assembly, the prototype in this study is assembled in a direct-driving way, as shown in Fig 1(D), to verify the inherent performance of the mechanism to ensure that the mechanical design of the proposed prosthesis has enough capacity for different users. Specifically, no UPE is assembled in the prototype. Besides, the stiffness of the SE is much higher than the optimal value (about two times). Considering the subject’s body weight, she almost cannot compress the SE during walking unless she uses the toe of the prosthesis to support the whole-body weight. Therefore, the connection between the output of the cable drive and the foot part can be regarded as an approximately rigid one. A simple and direct position control system is used to control the output position of the motor instead of the angular displacement of the ankle joint. In the control process, the ankle joint angle is converted to the position of the motor before the transmission as the control target. The feedback for the control system is also obtained directly from the motor. In this study, the control target is converted from the right ankle joint angular displacement result of the abovementioned first trial.

After preparing the prototype of the proposed device, the subject is asked to complete a tentative and off-the-recording trial, where she attempts to walk with wearing the device via a designed bypass adaptor (S1 Fig) and with the help of sticks. The sticks used in the trials are a pair of walking poles whose length can be adjusted between 90 to 110 cm (S2 Fig). During the attempt, the subject found an easy way to use the poles to keep herself balanced. In brief, both poles are operated to touch the ground to support the body weight simultaneously when the affected side of the lower limb, which is wearing the prosthesis, is in the stance phase of the gait cycle. The diagram of typical motion in one gait cycle of walking with sticks is shown in Fig 2(B), where the affected-side segments are blue.

Therefore, in the second trial, the subject is asked to walk with sticks without wearing the prosthesis at a slow, self-selected speed. According to her height, the walking poles are adjusted to 105 cm. The results of the second trial provide a reference for analysing the influence of using sticks on lower-limb biomechanics. For easy description, the walking pattern is termed normal walking with sticks (NWS) in this study.

**Fig 3:**
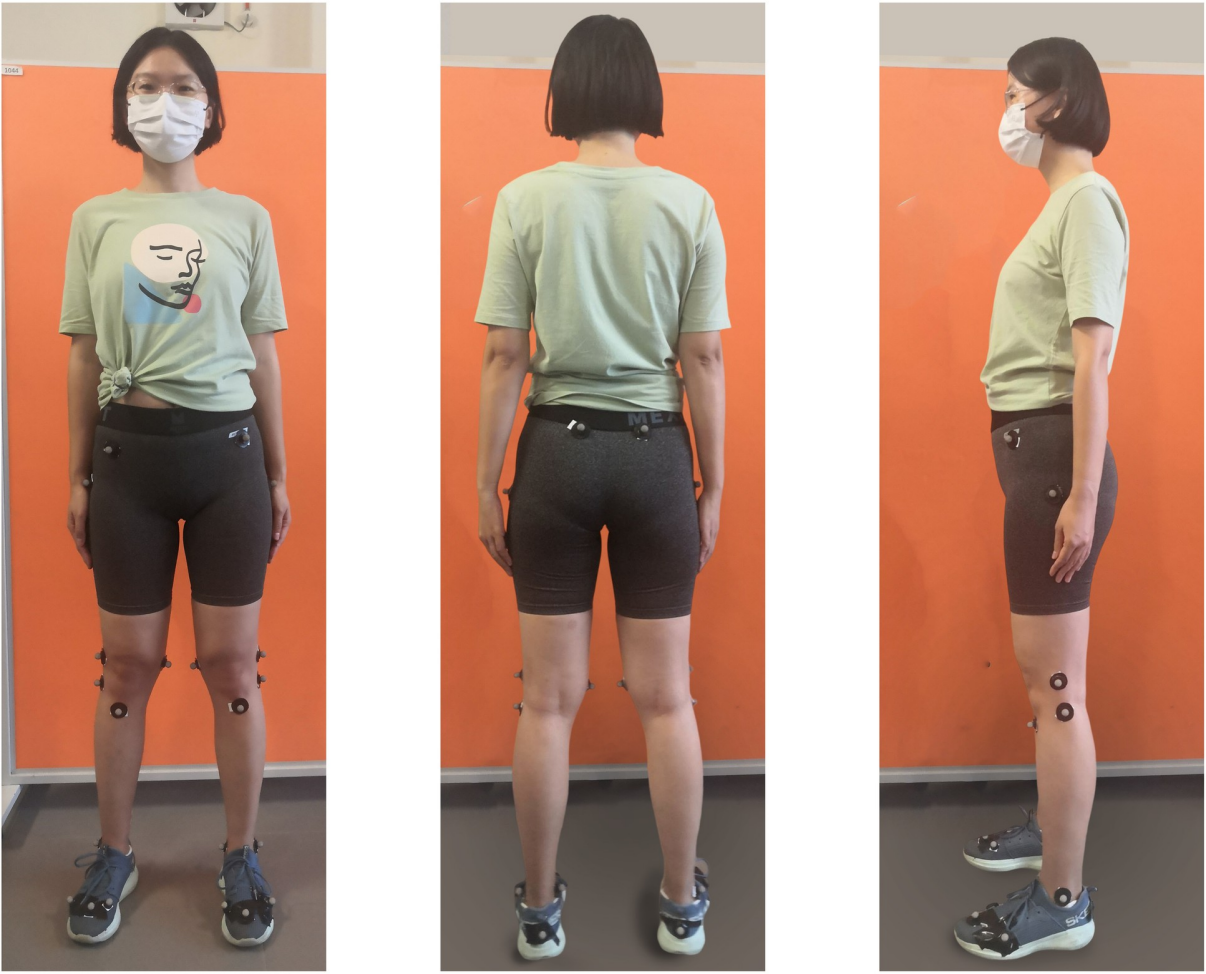
Placement of reflective markers of the optic motion capture system in the first and second trials.

In the first and second trials, the reflective markers of the optic motion capture system are stuck on the lower limbs of the subject (shown in Fig 3) according to the marker set guidelines [31]. Each side lower limb has 13 markers (ilium crest tubercle, ilium posterior superior, femur greater trochanter, femur lateral epicondyle, femur medial epicondyle, tibial tuberosity, apex of the styloid process, fibula apex of lateral malleolus, tibia apex of medial malleolus, posterior surface of calcaneus, proximal medial phalanx, head of second metatarsus, and head of fifth metatarsus). Thereinto, the markers on the right/left femur medial epicondyle and right/left tibia apex of medial malleolus are removed after the segment definition lest they have an influence on walking.

In the third and fourth trials, the subject is asked to walk with wearing the proposed powered ankle–foot prosthesis via the abovementioned bypass adaptor on the right side. The bypass adaptor is designed based on a walking boot, which is usually used to protect the foot and ankle joint from different kinds of injuries such as a sprain, shin splint, heel spurs, plantar fasciitis, and broken foot. As shown in Fig 1(E), a pylon is fixed on the walking boot via two 3D-printed linkers by screws. The assembly position of the pylon is low to ensure that the ankle joint of the powered ankle–foot prosthesis is lower than the bottom of the walking boot. In this case, the subject’s foot, which is wearing the bypass adaptor, will not touch the ground during walking with the prosthesis. In addition, the walking boot can fix the motion of the ankle joint and foot. Therefore, it also ensures that there is no motion of the subject’s ankle joint to disturb the operation of the powered ankle–foot prosthesis. Meanwhile, in order to guarantee the same length of two legs, several pieces of thick soles are fixed under the left shoe.

**Fig 4:**
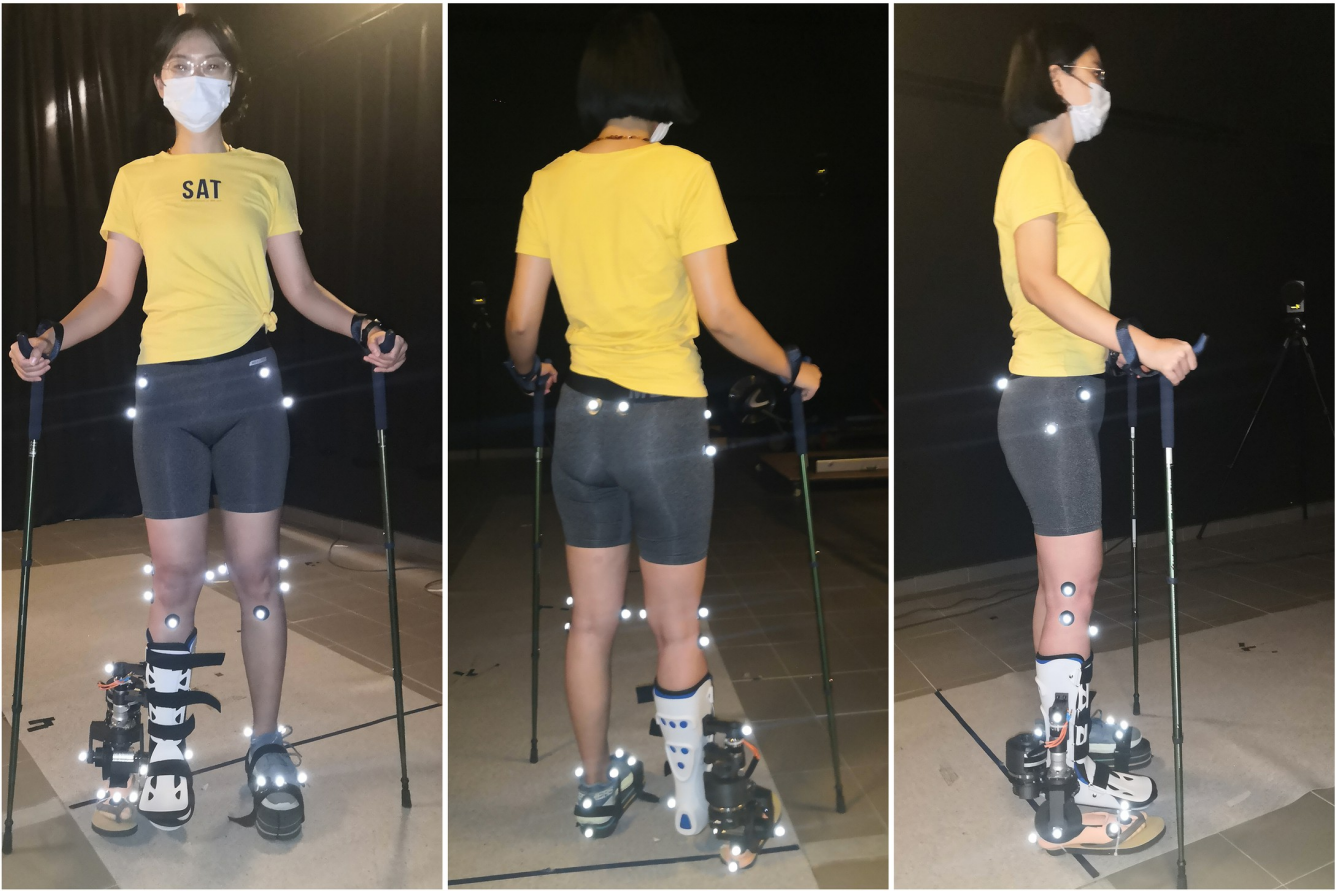
Placement of reflective markers of the optic motion capture system in the third and fourth trials.

In the third trial, the powered ankle–foot prosthesis does not connect to a power supply. Because of the abovementioned approximately rigid connection in the prototype, it is equivalent to the subject walking with the original ESAR prosthesis. Therefore, the third trial is termed walking with passive prosthesis (WPP) in this study. In the fourth trial, the prosthesis is powered by a DC power supply. During the fourth trial, the researcher controlled the DC power supply. That is, the researcher will power off the prosthesis when necessary lest the subject gets injured. The subject walks with the active mode of the prosthesis, which is termed walking with active prosthesis (WAP). Both the third and fourth trials are conducted after about 10 minutes of acclimatisation training. In the third and fourth trials, the method of using the sticks is the same as that in the second trial, but the length of the hiking poles is adjusted to 110 cm. The markers are placed on the lower limb and the prosthesis (shown in Fig 4), following the same marker set guidelines as used in the first and second trials.

Based on the above experiment setting, the affected and unaffected sides in this study are the right and left sides, respectively. The lower-limb GRF, kinematic, and kinetic results in the sagittal plane obtained from the second, third, and fourth trials will be compared with the counterparts from the first trial to investigate the influence of using the walking sticks, wearing the ESAR foot, and wearing the powered prosthesis on the subject’s lower-limb biomechanics, respectively. It is worth noting that the right ankle moment is not included. This study calculated the statistics for each trial’s results above. From the first to the third trials, three sets of biomechanical results for affected and unaffected sides are randomly selected for the statistics, whilst the number for the fourth trial statistics is 15. In the comparison, the correlation coefficient (CC) between the results of the second, third, and fourth trials and the first trial’s results are calculated to reflect the shape similarity of the two curves. Root-mean-square error (RMSE) is calculated to measure the differences between values.

## Results

The stride characteristics of the four trials are listed in Table 1. The results are presented in the format of average values accompanied by standard deviation in parentheses. Because of wearing the different prostheses via the bypass system, the height and body weight of the same subject in the third and fourth trials increased to 1.83 m and 68 kg, respectively. In all four trials, the walking speeds self-selected by the subject are around 0.75 m/s. At this speed, the differences in gait cycles and stance phases on both sides between NW and NWS are not significant. The gait cycles on both sides of WPP show a prolongation of about 0.3 s in comparison with those of NW. The prolongation of gait cycles from WAP is reduced, which is only 0.1 s longer than those of NW. The left stance phases of WPP and WAP are 9.13% and 5.23% more than that of NW, respectively. However, the stance phases on the right vary little. The double support phases on both sides of WPP and WAP occupy more percentages than those of NW in one gait cycle, and the increased percentage of WPP is more than 5%.

**Table 1:** Stride characteristics in four trials.

Items	NW	NWS	WPP	WAP
Height (m)	1.75		1.83	
Weight (kg)	64.5		68.0	
Length of Walking Sticks (m)	N/A	1.05	1.1	1.1
Speed (m/s)	0.75 (0.103)			
Left gait cycle (s)	1.35 (0.058)	1.36 (0.051)	1.63 (0.128)	1.46 (0.050)
Right gait cycle (s)	1.36 (0.053)	1.33 (0.024)	1.66 (0.144)	1.46 (0.059)
Left stance phase (%)	68.02 (1.170)	67.46 (1.459)	77.15 (1.952)	73.25 (0.662)
Right stance phase (%)	68.67 (0.714)	66.76 (1.348)	68.81 (2.786)	67.82 (2.357)
Left double support (%)	25.42 (0.598)	27.20 (1.398)	30.62 (1.968)	30.46 (1.607)
Right double support (%)	28.18 (1.422)	23.12 (1.140)	33.77 (1.888)	32.73 (2.507)

**Fig 5:**
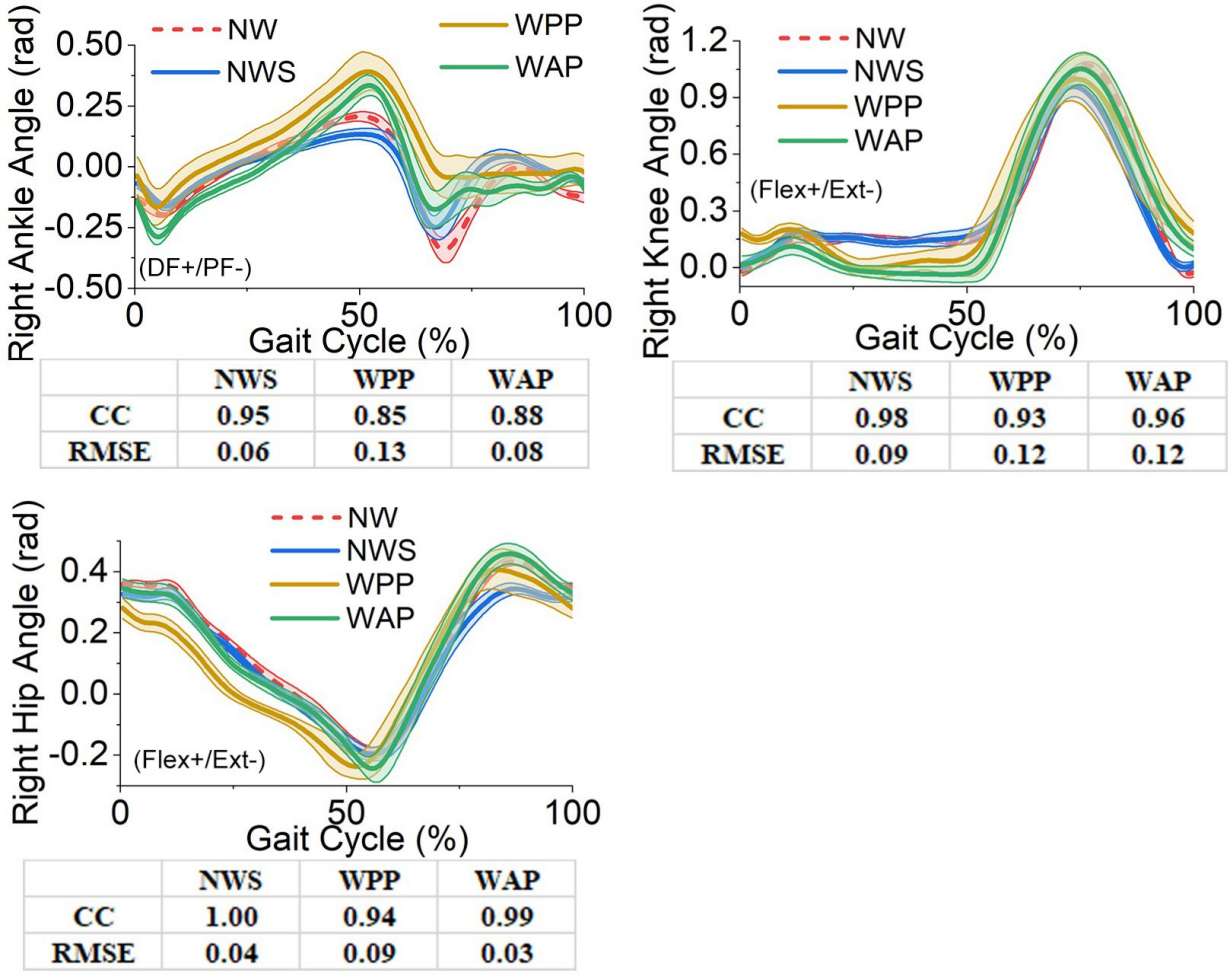
Lower-limb joints’ angles in the sagittal plane on the affected side. (a) right ankle angle; (b) right knee angle; (c) right hip angle. DF: dorsiflexion, PF: plantarflexion, Flex: flexion, Ext: extension.

**Fig 6:**
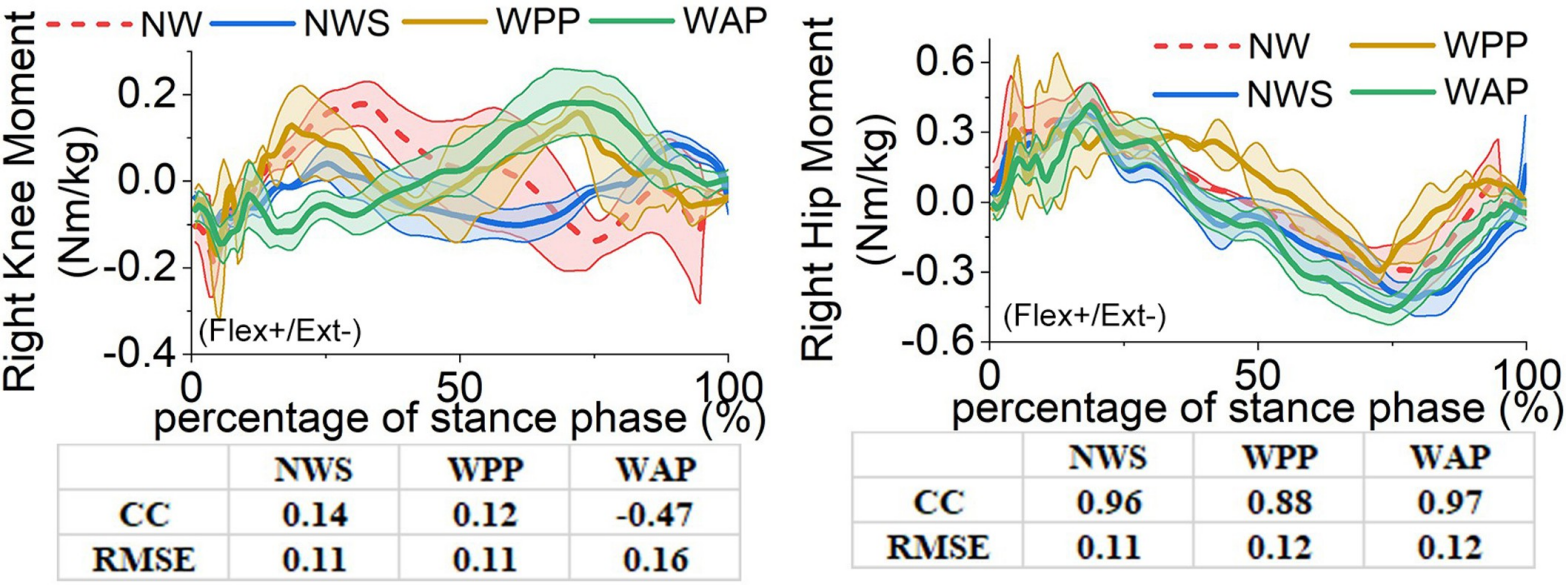
Lower-limb joints’ moments in the sagittal plane on the affected side. (a) right knee moment; (b) right hip moment. DF: dorsiflexion, PF: plantarflexion, Flex: flexion, Ext: extension.

**Fig 7:**
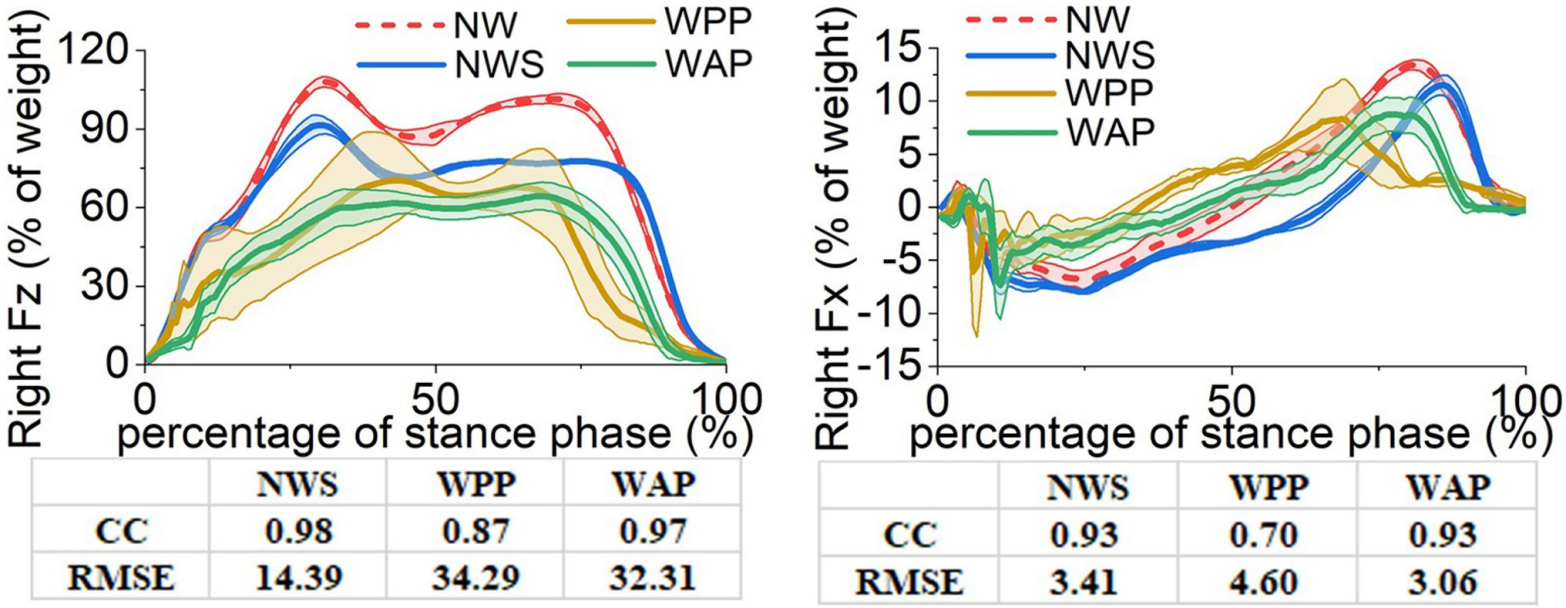
Ground reaction forces in the sagittal plane on the affected side. (a) component along the Z axis (b) component along the X axis.

**Fig 8:**
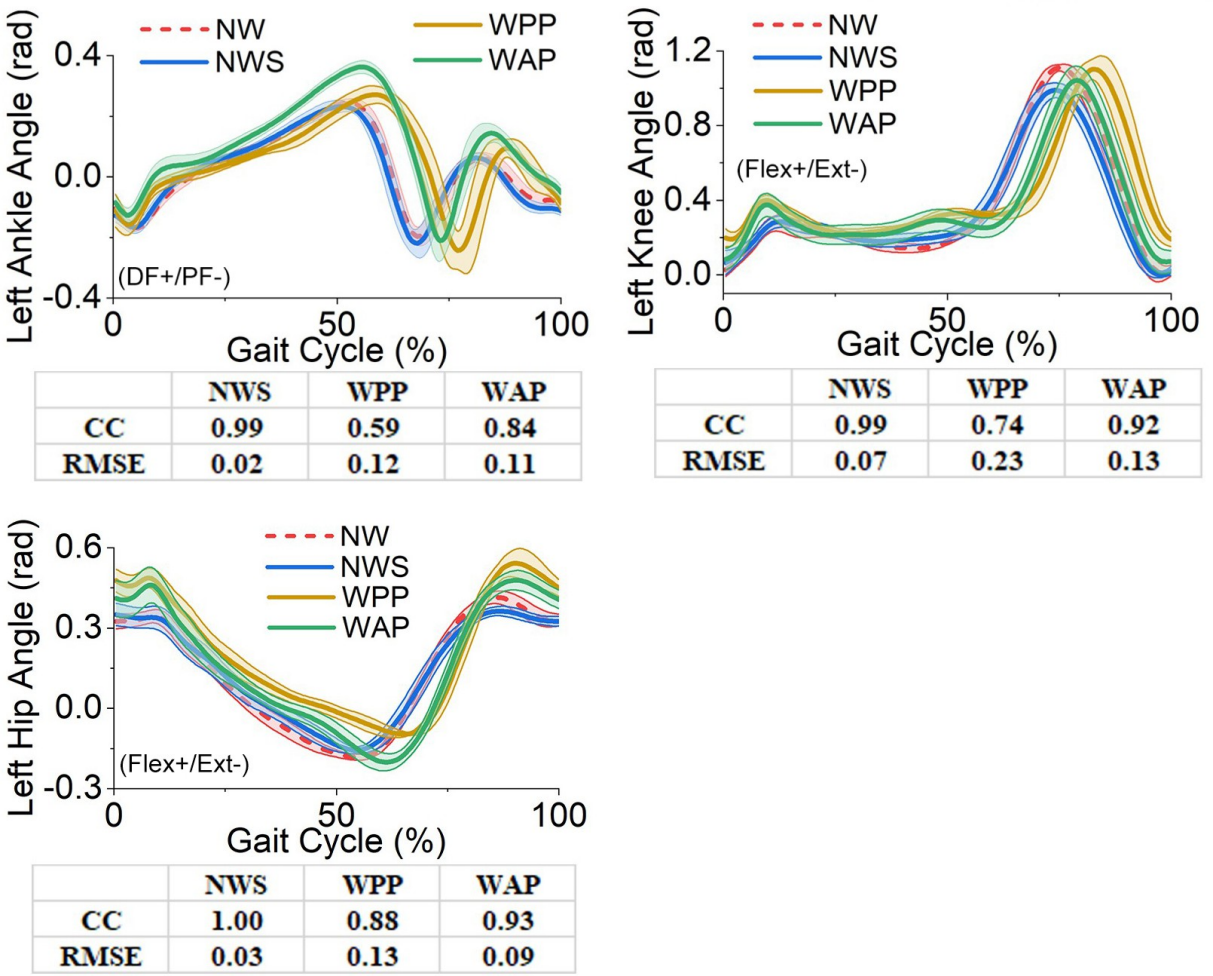
Lower-limb joints’ angles in the sagittal plane on the unaffected side. (a) left ankle angle; (b) left knee angle; (c) left hip angle. DF: dorsiflexion, PF: plantarflexion, Flex: flexion, Ext: extension.

**Fig 9:**
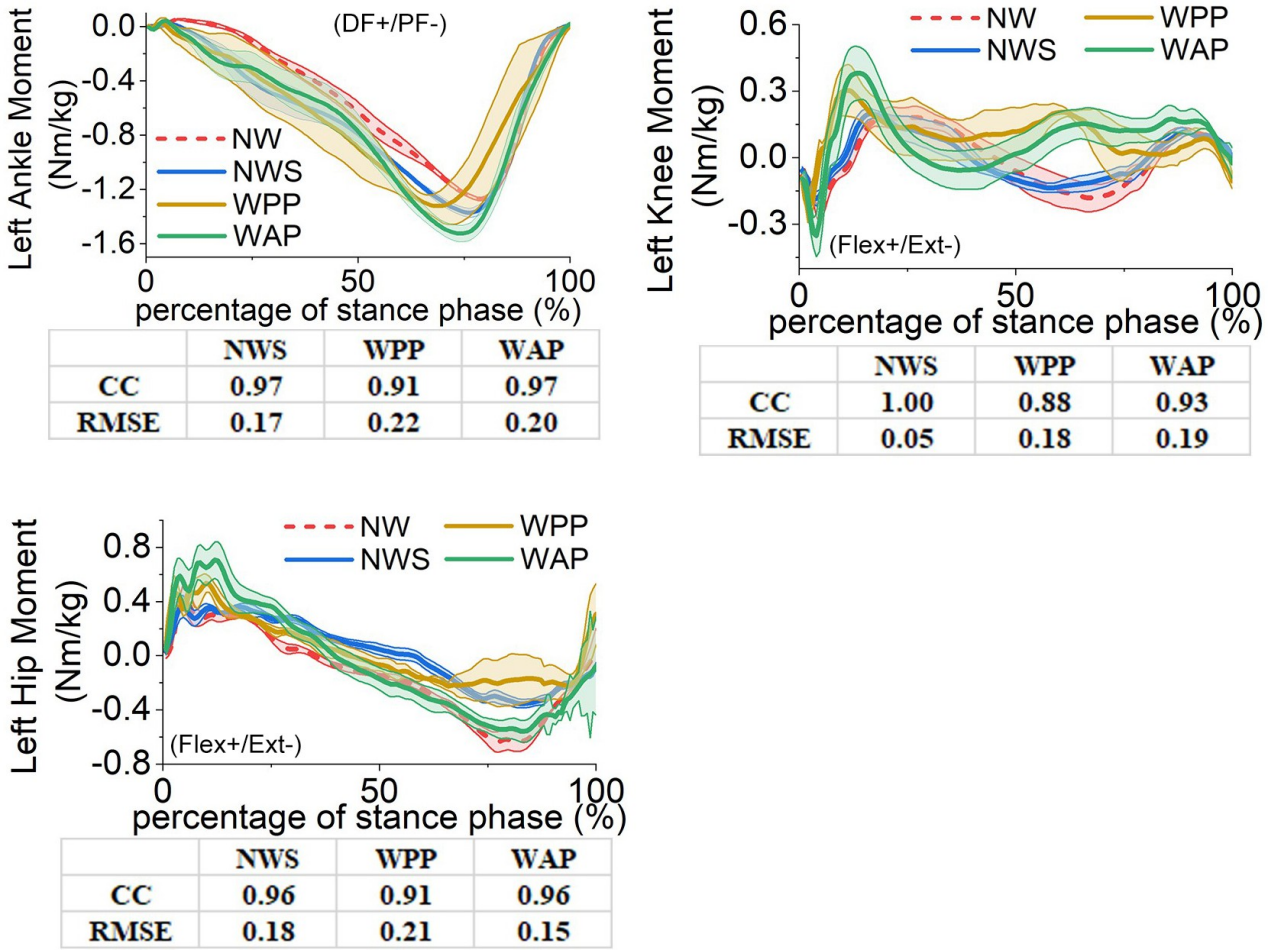
Lower-limb joints’ moments in the sagittal plane on the unaffected side. (a) left ankle moment; (b) left knee moment; (c) left hip moment. DF: dorsiflexion, PF: plantarflexion, Flex: flexion, Ext: extension.

**Fig 10:**
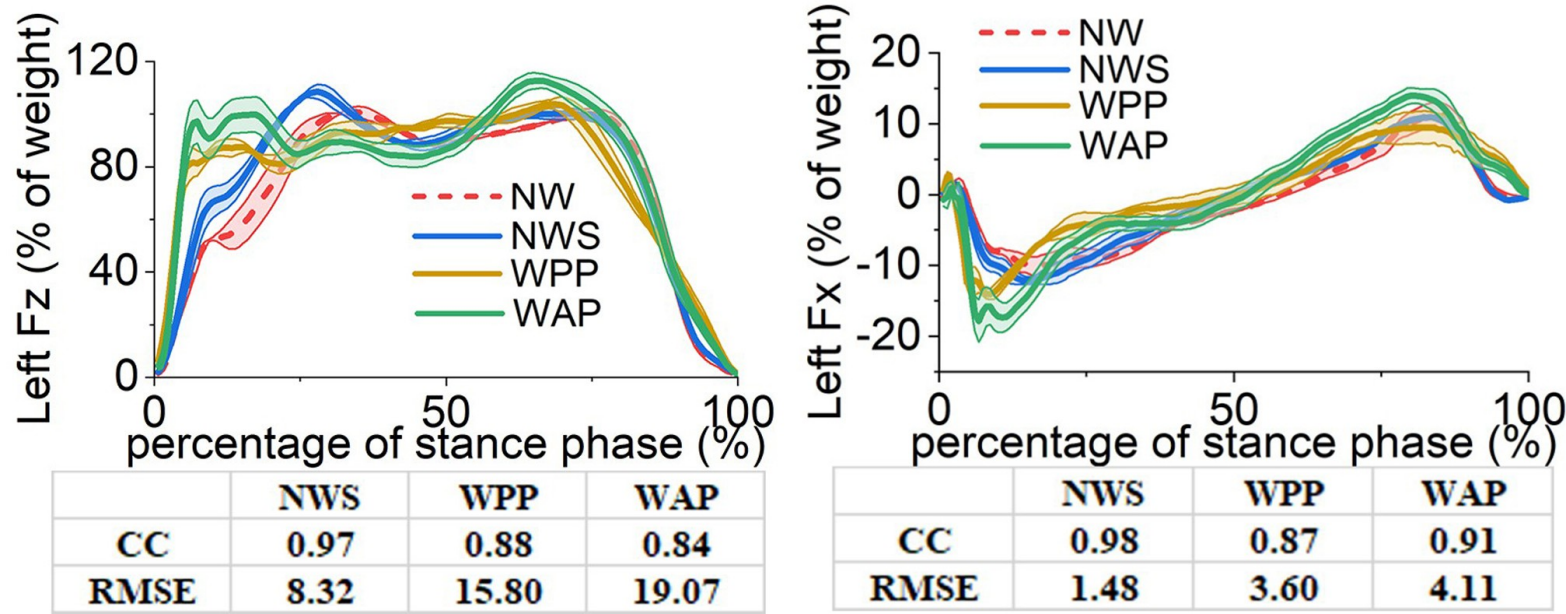
Ground reaction forces in the sagittal plane on the unaffected side. (a) component along the Z axis (b) component along the X axis.

The affected- and unaffected-side lower-limb joints’ angles, moments, and the GRF components in the sagittal plane obtained from the four trials are illustrated in Figs 5–10. All the results are in the format of average values plus/minus one standard deviation. Thereinto, the joints’ angles are averaged based on the entire gait cycle. However, the joints’ moments and GRF components are averaged based on the stance phase of the gait cycle.

The angular displacement of the right ankle joint directly reflects the difference between the ESAR and powered ankle-foot prostheses. As a reference, it can be found from comparing NWS and NW that using the walking sticks lightly reduces the range of dorsiflexion and plantarflexion of the right ankle joint from [–0.34, 0.21] rad to [–0.25, 0.13] rad. However, the outputs of wearing the ESAR prosthesis (WPP) and the designed powered prosthesis (WAP) have a larger peak in dorsiflexion, which are 0.39 rad and 0.33 rad, respectively, than that of NW. The noteworthy feature is the plantarflexion before the toe-off. The ESAR prosthesis can only recover from the dorsiflexion to the equilibrium position (around the zero point of the ankle joint) instead of providing further plantarflexion. However, the designed powered prosthesis can provide the plantarflexion with a peak value of –0.17 rad. From the overall perspective, WAP has a larger correlation coefficient and less RMSE than WPP with respect to NW. Thereinto, the RMSE of WAP is 0.08 rad, which is 61.54% of that of WPP.

Regarding the angles of other lower-limb joints, the influence of using the walking sticks in NWS is slight. Comparing the curves of NWS with those of NW, all the values of the correlation coefficient are more than 0.98, and those of RMSE are less than 0.1 rad. However, the effects of WPP and WAP are more significant than those of NWS, and they are listed as follows.

For the right knee joint, the greatest influence of WPP and WAP is that the joint angle is decreased in the period of 10% to 50% gait cycle. Both WPP and WAP also lightly bring forward the percentage of the gait cycle when the peak angle of the right knee joint happens. Compared with the peak value of WPP (1.00 rad), that of WAP (1.05 rad) is more similar to that of NW (1.07 rad). In addition, WAP also has a larger correlation coefficient and less RMSE than WPP.

For the right hip joint, WPP and WAP have a negative angular difference with respect to NW in the first 50% gait cycle, and the difference between NW and WPP is much larger than that between NW and WAP. In addition, WPP obviously shortens the percentage of the gait cycle in which the peak angle of hip extension (in negative) happens, about 4.5%. The peak angle of hip extension increases by 0.44 rad (in negative) in WAP. However, in the entire gait cycle, the difference between WAP and NW is not significant. With respect to NW, WAP has a correlation coefficient of 0.99, which is higher than that of WPP, and the RMSE of WAP is less than one-third of that of WPP.

For the left ankle joint, WAP and WPP have significant influences on its kinematics. They prolong the stance phase, but the extent of WAP is lighter than that of WPP. Therefore, the correlation coefficient of WAP is 0.25 higher than that of WPP. WAP and WPP also increase the maximum angle of dorsiflexion in the stance phase. Although the peak angles of WAP and WPP are 0.36 rad and 0.27 rad, respectively, the RMSE of WAP (0.11 rad) in the entire gait cycle is still lower than that of WPP (0.12 rad).

For the left knee and hip joints, because of the prolongation of the stance phase in the left ankle joint’s kinematics, with respect to NW, the peak angle of knee flexion and that of hip extension (in negative) in WAP and WPP appear later correspondingly. Thereinto, the influence of WAP is lighter than that of WPP. Therefore, WAP performs better in the correlation coefficient and RMSE than WPP.

On the aspect of GRF, this study focuses on the data in the sagittal plane, so only the components of the GRF along the Z-axis and X-axis (***F***_***Z***_ and ***F***_***X***_) are reported. In addition, as wearing the prosthesis increases the subject’s body weight in the third and fourth trials, the force results are normalised with respect to the body weights.

For the right ***F***_***Z***_, using the walking sticks allows the subject to transfer a part of the body weight to the sticks, which results in a decrease in value. In NWS, the decrease of the right ***F***_***Z***_, which is equal to about 15% of the body weight, happens in the middle stage of the gait cycle. However, the decrease at the beginning and ending stages of the gait cycle is not obvious. In WPP and WAP, the decrease of the values happens in the entire gait cycle, and the extents are more considerable, which means that the subject puts more body weight on the sticks. The considerably decreased values result in large RMSE values (34.29 in WPP and 32.31 in WAP in the unit of % of weight). From the perspective of the correlation coefficient and RMSE, the influence of WAP is less than that of WPP in comparison with NW.

For the right ***F***_***X***_, during the period from the 40% gait cycle to the positive peak value occurrence, using the walking sticks (NWS) leads to a delay in its value change from negative to positive. However, in WPP and WAP, wearing the ESAR foot and the designed powered prostheses makes the percentage of the direction change of the right ***F***_***X***_ shift forward (at about 34.5% of the gait cycle in WPP and about 44.0% in WAP). In addition, WPP and WAP also cause a rapid variation of the values in the first 10% gait cycle and influence the range of the right ***F***_***X***_ values. Overall, WAP has a larger correlation coefficient and less RMSE than WPP.

For the left ***F***_***Z***_, using the walking sticks in NWS causes a shift forward of the curve during the 10% to 80% gait cycle. In the curves of WPP and WAP, the values of the left ***F***_***Z***_ increase more rapidly in the initial stage of the gait cycle than in NW, which means that the subject transfers the body weight to the left side earlier after the left heel strike. The curve of WAP remains the two-peak shape with a larger second-peak value, which is 112.70% of the subject’s body weight. However, the curve of WPP does not show the first-peak value. From the perspective of the correlation coefficient and the values of RMSE, WAP has a more significant influence on the left ***F***_***Z***_ than WPP with respect to NW.

For the left ***F***_***X***_, using the walking sticks in NWS does not have a significant influence. A similar rapid increase in the initial stage of the gait cycle also happens to the values in the negative direction in WPP and WAP. On the aspect of the peak value in positive, WAP and WPP have a larger value (13.99% of the subject’s body weight) and a smaller one (9.53%) than that of NW (11.29%), respectively. In addition, WAP worsens the peak value in negative more significantly than WPP. Therefore, with respect to NW, the RMSE value of WAP is larger than that of WPP. However, the correlation coefficient of WAP performs better than WPP.

Finally, the influence of different walking conditions on the kinetics of lower-limb joints, except for the right ankle joint, can be analysed. The right knee moment is most considerably affected by three different walking conditions. Using the walking sticks in NWS causes the curve shape of the right knee moment to change dramatically. With respect to NW, the correlation coefficient of the two curves is less than 0.2. A similar situation happens to WPP. The correlation coefficient between the curves of WAP and NW is even negative. The RMSE values of NW, WPP, and WAP are more than 0.1 Nm/kg, which is very large considering the range of the knee moment during NW within –0.2 to 0.2 Nm/kg.

The influences on the other four lower-limb joints’ moments are not as considerable as that on the right knee moment. For the right hip moment, NWS and WAP make the peak moment in the negative direction larger, from –0.29 Nm/kg in NW to –0.41 Nm/kg and –0.47 Nm/kg, respectively. However, their correlation coefficient values with respect to NW, which are 0.96 and 0.97, respectively, are much better than that of WPP (0.89). The values of the RMSE from WAP and WPP to NW are the same (0.12 Nm/kg).

For the left ankle moment, both using the walking sticks and wearing the prostheses increase the absolute value of the moment during the 10% to 80% of the stance phase. The minimums of the moment in four trials are –1.27 Nm/kg (NW), –1.37 Nm/kg (NWS), –1.32 Nm/kg (WPP), and –1.53 Nm/kg (WAP), respectively. However, in comparison with NW, WAP has a larger correlation coefficient and less RMSE than WPP.

For the left knee moment, using the walking sticks in NWS does not have a significant influence on it. The influence of wearing the ESAR and powered prostheses in WPP and WAP possesses two main features. One is that, in the initial stage of the stance phase, the joint moments have a more rapid change and a larger amplitude than NW. The other is that, during 50% to 70% of the stance phase, a direction change of the moment occurs. Overall, WAP achieves a higher correlation coefficient than WPP. However, the RMSE of WAP (0.19 Nm/kg) is slightly larger than that of WPP (0.18 Nm/kg).

For the left hip moment, the joint moment obtained from NWS has a larger value in negative than from NW after about 30% of the stance phase. In the initial stage of the stance phase, the maximums of the moment in WPP (0.54 Nm/kg) and WAP (0.71 Nm/kg) are larger than that of NW (0.44 Nm/kg). During the 70% to 90% gait cycle, the absolute value of the joint moment in WPP is significantly less than that in NW. However, the similarity between WAP and NW is high. Therefore, WAP performs better in the correlation coefficient and RMSE than WPP with respect to NW.

## Discussion

Based on a novel powered ankle-foot prosthesis, this study compared the influence of wearing the ESAR and powered ankle–foot prostheses for a rookie on the lower-limb biomechanics in the sagittal plane during walking on level ground to evaluate the novel prosthesis. Different from the results of highly active users who received intensive rehabilitation training in the literature [22–25], the influence on affected and unaffected lower-limb biomechanics when the rookie wearing the transtibial prosthesis is considerable, as expected. The differences between the powered and ESAR prostheses’ influences also exist. It is suggested that related professionals pay attention to these influences, which may contribute to the necessary tuning of adaptive training. In addition, the non-amputated subject is employed, considering comparability. Although it is common to use a comparative method between the amputees and control subjects who are matched to the amputees in terms of age, gender, height, and weight, the comparison is not perfect since each individual has a specific gait. This problem can be solved by comparing with the non-amputated subject’s own normal gait. The non-amputated subject recruited in this study has never been trained to use a prosthesis or other similar skills. Therefore, she can be regarded as a rookie user with moderate or high levels of activity.

The analysis of different aspects of the results contributes to a complete understanding of the influence of wearing different prostheses on lower-limb biomechanics. From the perspective of stride characteristics, the average gait cycles on both sides, when the subject wears the prostheses, are prolonged significantly. Considering the average walking speeds are similar in four trials, it implies that the subject has to increase the stride length to achieve the pace that she is used to. In this case, it can also be inferred that the subject will cost more energy than the normal walking. Similarly, the left stance phase is also prolonged significantly, but the right stance phase does not have an obvious change in comparison with NW, which means the subject needs more support from the left (unaffected) side to keep balanced as the prosthesis is worn on the right. Based on the prolongation of the left stance phase, the prolongation of both the left and right double support phases can be explained. The left double support phase (from the right heel strike to the left toe-off) is prolonged because the subject tends to delay the left toe-off, and the prolongation of the right double support phase is because the subject needs an earlier entrance to the left stance phase in the right swing phase. Regarding the aforementioned three aspects of the stride characteristics, the prolongation extents of WAP are less than those of WPP with respect to NW, which demonstrates that wearing the powered ankle-foot prosthesis is instrumental to remitting the subject’s high energy consumption and keeping balanced during walking.

Regarding kinematics, the difference in the angle of the affected ankle joint between wearing the prostheses and normal walking is the most noteworthy, which may be the reason for the differences in other lower-limb joints’ kinematics and kinetics. The ESAR and powered prostheses show common features––a larger peak angle of dorsiflexion and a less peak angle of plantarflexion. Thereinto, in comparison with WPP, the most important improvement of WAP is to provide further plantarflexion before the toe-off, where the ankle joint displacement can exceed the initial equilibrium position of the prosthesis. Therefore, the proposed powered device can provide further propulsive power for the lifting of the human body’s centre of gravity while walking on level ground, which contributes to the recovery of amputees’ gait, especially for rookie amputees. For other joints’ kinematics, on the affected side, wearing the prostheses makes the peak angle of knee and hip extension larger. However, the difference between WPP and WAP is not considerable. On the unaffected side, both WPP and WAP enlarge the peak angle of the ankle dorsiflexion and hip flexion, and wearing the powered prosthesis has a greater influence. Therefore, it is worth noting that extra fatigue or soreness may happen to the muscles around the hip and ankle joints on the unaffected side when rookie users wear powered ankle-foot prostheses.

On the aspect of GRF and joints’ moments, including the influence of using the walking sticks, the greatest influence of wearing the prostheses happens to the knee joint on the affected side. In the NWS, WPP, and WAP results, the curve’s trend experiences significant influence, yet the maximum and minimum knee moment remains comparable to that of NW. The reason for the change in the curve trend should be that the variation of the subject’s mass centre is different from that of NW because the subject transfers some body weight to the walking sticks. However, it is still unclear whether the change of the knee moment’s curve trend during walking will influence the knee joint. Therefore, for transtibial amputees who are rookies to wear prostheses, the healthy situation of the knee joint on the affected side is of great concern. According to the Z-axis GRF component on the left, the premature transfer of body weight to the unaffected side also demonstrates that the subject relies more on the unaffected leg to keep balanced, which is consistent with the analysis of the stride characteristics. In addition, the peak moments of ankle, knee, and hip joints on the unaffected side are increased by wearing the prostheses, especially by wearing the powered prosthesis. Therefore, it may be a serious problem for the rookies to feel uncomfortable on the unaffected side. However, with enough training, the users of transtibial prostheses should be more adaptable to the device. Hence, the gait will tend to be symmetrical, and the influence on the unaffected leg will be minimised.

Although the influence of using the walking sticks is only regarded as the reference for other results instead of the emphasis of this study, it also provides some inspiration. The most significant influence of using the walking sticks with the method mentioned in this study is to decrease the vertical component of GRF on the supported side, which may be instrumental in reducing the compression from the socket of the prosthesis to the residual limb. However, as abovementioned, it also worsens the knee situation on the affected side. Therefore, it is important to use the auxiliary method correctly and appropriately.

From the perspective of the similarity to normal walking, the statistics among the total 15 groups of kinematic and kinetic results can be used to evaluate the designed powered ankle-foot prosthesis. In 10 groups, WAP has higher correlation coefficients and lower RMSEs than WPP. In 3 groups, WAP has higher correlation coefficients than WPP and similar RMSEs to WPP. Only in 2 groups the performance of WAP is slightly worse than that of WPP. Therefore, it demonstrates that it is easier for the rookies to recover their normal walking gait by wearing the designed powered ankle-foot prosthesis than by wearing the ESAR foot. It is also worth noting that, as abovementioned, the prototype used in this study is assembled in a direct-driving way with a simple position control system. That is, the benefits of elastic elements and control system are minimised. Compared with the ESAR foot, the only benefit of the powered ankle–foot prosthesis is the input of net positive power, which has made for better performance and slighter influence. Therefore, it is believed that the performance of the designed powered ankle-foot prosthesis can be improved further after applying the optimal assembly and advanced control system based on biomechanical estimation technology [32, 33], while the undesirable influence on the users’ lower-limb biomechanics can also be reduced. The above results and discussion also imply that, if conditions allow, amputees unfamiliar with using the ankle–foot prosthesis can start with the powered ankle–foot prosthesis rather than the ESAR foot.

Finally, this study has some limitations. As this study serves as a pilot investigation for the preliminary verification of a novel powered ankle–foot prosthesis, only one subject was recruited for the trials. Therefore, the results presented in this study provide preliminary, but not general, evidence about the effect of wearing different prostheses on lower-limb biomechanics. In future, more subjects, including non-amputated and amputated ones, will be recruited for further trials. More daily motions, such as stairs up/down and slope ascending/descending, will be investigated. Biomechanics analysis will not be limited to lower limbs but expanded to the whole trunk.

## Conclusions

This study reported a novel powered ankle–foot prosthesis and completed the preliminary verification of the prototype. The influence of wearing the novel powered ankle-foot and ESAR prostheses on lower-limb biomechanics based on a rookie walking on level ground is investigated. A non-amputated subject, who can be regarded as a rookie, is recruited in this study. The results of lower-limb joints’ angle, moments, and GRF components in the sagittal plane are compared, which shows that wearing different prostheses has an influence on both the affected- and unaffected-side lower limbs. However, overall, wearing the novel powered ankle–foot prosthesis has less influence than wearing the ESAR prosthesis.

## Supporting information

S1 Fig Bypass adaptor based on a walking boot. (TIF)

S2 Fig Adjustable hiking poles. (TIF)

S1 File Elastic elements’ optimisation method. (DOCX)

S2 File Original data of motion capture of four trials. (ZIP)

## References

[1] Soares ASOdC, Yamaguti EY, Mochizuki L, Amadio AC, Serrão JC. Biomechanical parameters of gait among transtibial amputees: a review. Sao Paulo Medical Journal. 2009;127. doi: 10.1590/s1516-31802009000500010 20169280 PMC11553117

[2] Chow DHK, Holmes AD, Lee CKL, Sin SW. The effect of prosthesis alignment on the symmetry of gait in subjects with unilateral transtibial amputation. Prosthetics and Orthotics International. 2006;30(2):114–28. doi: 10.1080/03093640600568617 16990222

[3] Eshraghi A, Abu Osman NA, Karimi M, Gholizadeh H, Soodmand E, Abas WABW. Gait biomechanics of individuals with transtibial amputation: effect of suspension system. PLOS ONE. 2014;9(5):e96988. doi: 10.1371/journal.pone.0096988 24865351 PMC4035274

[4] Boutwell E, Stine R, Hansen A, Tucker K, Gard S. Effect of prosthetic gel liner thickness on gait biomechanics and pressure distribution within the transtibial socket. Journal of Rehabilitation Research and Development. 2012;49(2):227–40. doi: 10.1682/jrrd.2010.06.0121 22773525

[5] Coleman KL, Boone DA, Smith DG, Czerniecki JM. Effect of trans‐tibial prosthesis pylon flexibility on ground reaction forces during gait. Prosthetics and Orthotics International. 2001;25(3):195–201. doi: 10.1080/03093640108726602 11860093

[6] Atallah H, Kenney L, Howard D, Liu A, Head J. Effects of a modified passive socket system on short-term changes in residuum volume and comfort: A preliminary study in transtibial amputees. Prosthetics and orthotics international. 2022;46(1):54–60. doi: 10.1097/PXR.0000000000000053 . 34772866

[7] Staros A. The SACH (solid-ankle cushion-heel) foot. Orthotics and Prosthetics. 1957;11(2):23–31.

[8] Össur. Pro-Flex® XC [Internet]. USA2024 [2024 March]. Available from: https://www.ossur.com/en-us/prosthetics/feet/pro-flex-xc.

[9] Ottobock. Restore Germany2024 [2024 March]. Available from: https://www.ottobock.com/en-us/product/VS5.

[10] Stevens PM, Rheinstein J, Wurdeman SR. Prosthetic foot selection for individuals with lower-limb amputation: a clinical practice guideline. Journal of Prosthetics and Orthotics. 2018;30(4):175–80. doi: 10.1097/JPO.0000000000000181 . 30473606 PMC6221375

[11] Klute GK, Czerniecki J, Hannaford B, editors. Development of powered prosthetic lower limb. Proceedings of the 1st National Meeting, Veterans Affairs Rehabilitation, Research and Development Service; 1998 1–3 Oct.; Washington, DC.

[12] Liu J, Abu Osman NA, Al Kouzbary M, Al Kouzbary H, Abd Razak NA, Shasmin HN, et al. Classification and comparison of mechanical design of powered ankle–foot prostheses for transtibial amputees developed in the 21st century: a systematic review. Journal of Medical Devices. 2021;15(1). doi: 10.1115/1.4049437

[13] Hood S, Gabert L, Lenzi T. Powered knee and ankle prosthesis with adaptive control enables climbing stairs with different stair heights, cadences, and gait patterns. IEEE Transactions on Robotics. 2022;38(3):1430–41. doi: 10.1109/TRO.2022.3152134 35686286 PMC9175645

[14] Persine S, Leteneur S, Gillet C, Bassement J, Charlaté F, Simoneau-Buessinger E. Kinetic adaptations of the intact limb in transfemoral amputees using a microprocessor prosthetic knee. Gait & Posture. 2024;108:170–6. doi: 10.1016/j.gaitpost.2023.11.022 38100955

[15] Tran M, Gabert L, Hood S, Lenzi T. A lightweight robotic leg prosthesis replicating the biomechanics of the knee, ankle, and toe joint. Science robotics. 2022;7(72):eabo3996. doi: 10.1126/scirobotics.abo3996 36417500 PMC9894662

[16] Lathouwers E, Díaz MA, Maricot A, Tassignon B, Cherelle C, Cherelle P, et al. Therapeutic benefits of lower limb prostheses: a systematic review. Journal of NeuroEngineering and Rehabilitation. 2023;20(1):4. doi: 10.1186/s12984-023-01128-5 36639655 PMC9840272

[17] Herr HM, Grabowski AM. Bionic ankle–foot prosthesis normalizes walking gait for persons with leg amputation. Proceedings of the Royal Society B: Biological Sciences. 2012;279(1728):457–64. doi: 10.1098/rspb.2011.1194 21752817 PMC3234569

[18] Russell Esposito E, Aldridge Whitehead JM, Wilken JM. Step-to-step transition work during level and inclined walking using passive and powered ankle–foot prostheses. Prosthetics and Orthotics International. 2016;40(3):311–9. doi: 10.1177/0309364614564021 . 25628378

[19] Gardinier ES, Kelly BM, Wensman J, Gates DH. A controlled clinical trial of a clinically-tuned powered ankle prosthesis in people with transtibial amputation. Clinical Rehabilitation. 2018;32(3):319–29. doi: 10.1177/0269215517723054 . 28750586

[20] D’Andrea S, Wilhelm N, Silverman AK, Grabowski AM. Does use of a powered ankle-foot prosthesis restore whole-body angular momentum during walking at different speeds? Clinical Orthopaedics and Related Research®. 2014;472(10):3044–54. doi: 10.1007/s11999-014-3647-1 24781926 PMC4160507

[21] Grabowski AM, D’Andrea S. Effects of a powered ankle-foot prosthesis on kinetic loading of the unaffected leg during level-ground walking. Journal of NeuroEngineering and Rehabilitation. 2013;10(1):49. doi: 10.1186/1743-0003-10-49 23758860 PMC3685554

[22] Ferris AE, Aldridge JM, Rábago CA, Wilken JM. Evaluation of a powered ankle-foot prosthetic system during walking. Archives of Physical Medicine and Rehabilitation. 2012;93(11):1911–8. doi: 10.1016/j.apmr.2012.06.009 22732369

[23] Rábago CA, Aldridge Whitehead J, Wilken JM. Evaluation of a powered ankle-foot prosthesis during slope ascent gait. PLOS ONE. 2016;11(12):e0166815. doi: 10.1371/journal.pone.0166815 27977681 PMC5157979

[24] Aldridge JM, Sturdy JT, Wilken JM. Stair ascent kinematics and kinetics with a powered lower leg system following transtibial amputation. Gait & Posture. 2012;36(2):291–5. doi: 10.1016/j.gaitpost.2012.03.013 22571821

[25] Gates DH, Aldridge JM, Wilken JM. Kinematic comparison of walking on uneven ground using powered and unpowered prostheses. Clinical Biomechanics. 2013;28(4):467–72. doi: 10.1016/j.clinbiomech.2013.03.005 23602128

[26] Au SK, Herr HM. Powered ankle-foot prosthesis. IEEE Robotics & Automation Magazine. 2008;15(3):52–9. doi: 10.1109/MRA.2008.927697

[27] Eilenberg MF, Geyer H, Herr H. Control of a powered ankle–foot prosthesis based on a neuromuscular model. IEEE Transactions on Neural Systems and Rehabilitation Engineering. 2010;18(2):164–73. doi: 10.1109/TNSRE.2009.2039620 20071268

[28] Markowitz J, Krishnaswamy P, Eilenberg MF, Endo K, Barnhart C, Herr H. Speed adaptation in a powered transtibial prosthesis controlled with a neuromuscular model. Philosophical Transactions of the Royal Society B: Biological Sciences. 2011;366(1570):1621–31. doi: 10.1098/rstb.2010.0347 21502131 PMC3130448

[29] Liu J, Abu Osman NA, Al Kouzbary M, Al Kouzbary H, Abd Razak NA, Shasmin HN, et al. Optimization and comparison of typical elastic actuators in powered ankle-foot prosthesis. International Journal of Control, Automation and Systems. 2022;20(1):232–42. doi: 10.1007/s12555-020-0980-x

[30] Liu J, Abu Osman NA, Al Kouzbary M, Al Kouzbary H, Abd Razak NA, Shasmin HN, et al. Stiffness estimation of planar spiral spring based on Gaussian process regression. Scientific Reports. 2022;12(1):11217. doi: 10.1038/s41598-022-15421-1 35780242 PMC9250535

[31] C-Motion. Marker Set Guidelines 2017 [2023 Jan]. Available from: https://www.c-motion.com/v3dwiki/index.php/Marker_Set_Guidelines.

[32] Mobarak R, Tigrini A, Verdini F, Al-Timemy AH, Fioretti S, Burattini L, et al. A minimal and multi-source recording setup for ankle joint kinematics estimation during walking using only proximal information from lower limb. IEEE Transactions on Neural Systems and Rehabilitation Engineering. 2024;32:812–21. doi: 10.1109/TNSRE.2024.3364976 38335075

[33] Kouzbary HA, Kouzbary MA, Tham LK, Liu J, Shasmin HN, Osman NAA. Generating an Adaptive and Robust Walking Pattern for a Prosthetic Ankle–Foot by Utilizing a Nonlinear Autoregressive Network With Exogenous Inputs. IEEE Transactions on Neural Networks and Learning Systems. 2022;33(11):6297–305. doi: 10.1109/TNNLS.2021.3076060 33979293

