# Supplementary figures and images for: Design and preliminary verification of a novel powered ankle–foot prosthesis: From the perspective of lower-limb biomechanics compared with ESAR foot
Source: PLoS One. 2024 Jun 7;19(6):e0303397. doi: 10.1371/journal.pone.0303397 (PMC11161064; doi:10.1371/journal.pone.0303397)

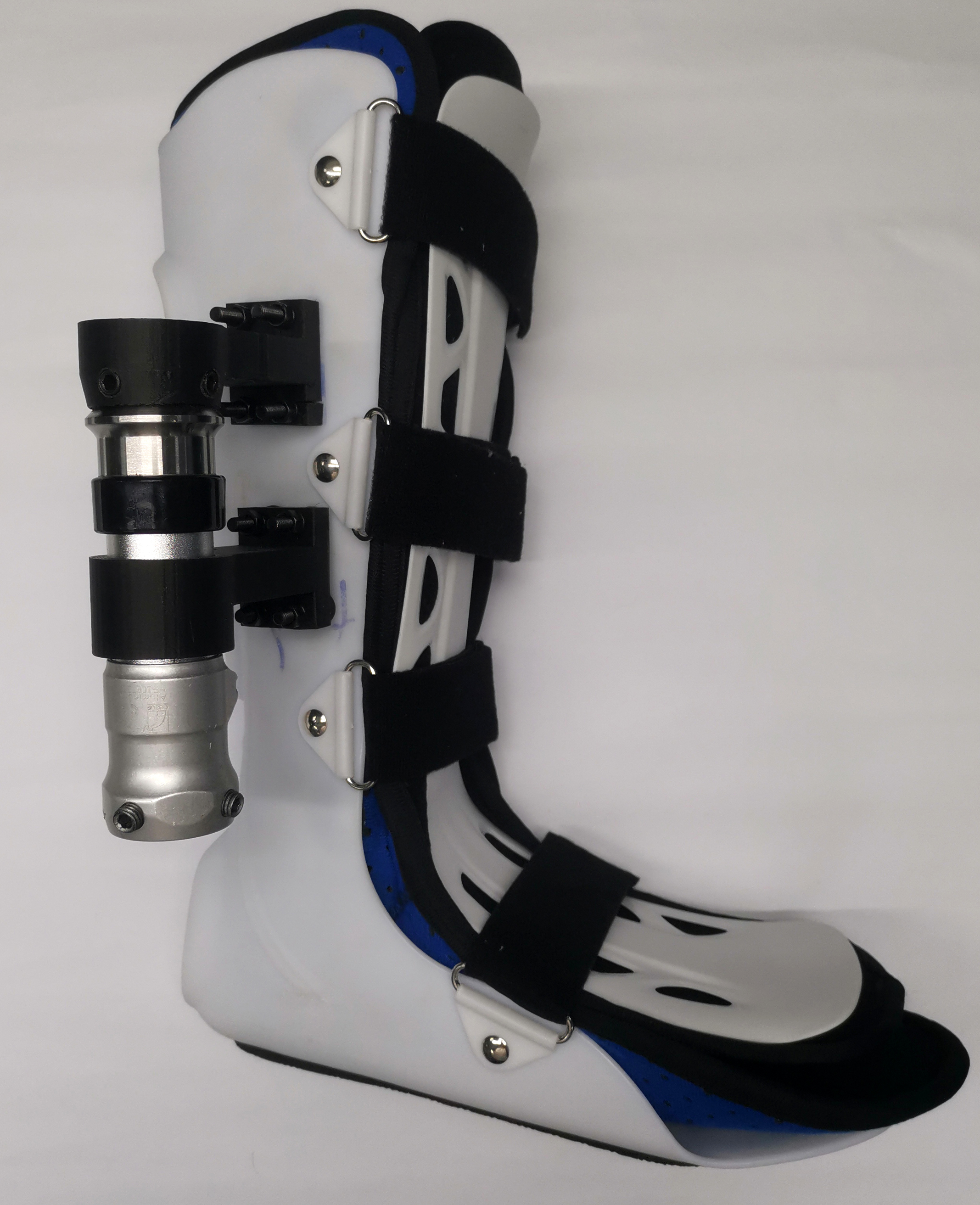

Supplement: S1 Fig — (TIF) [file pone.0303397.s001.tif]

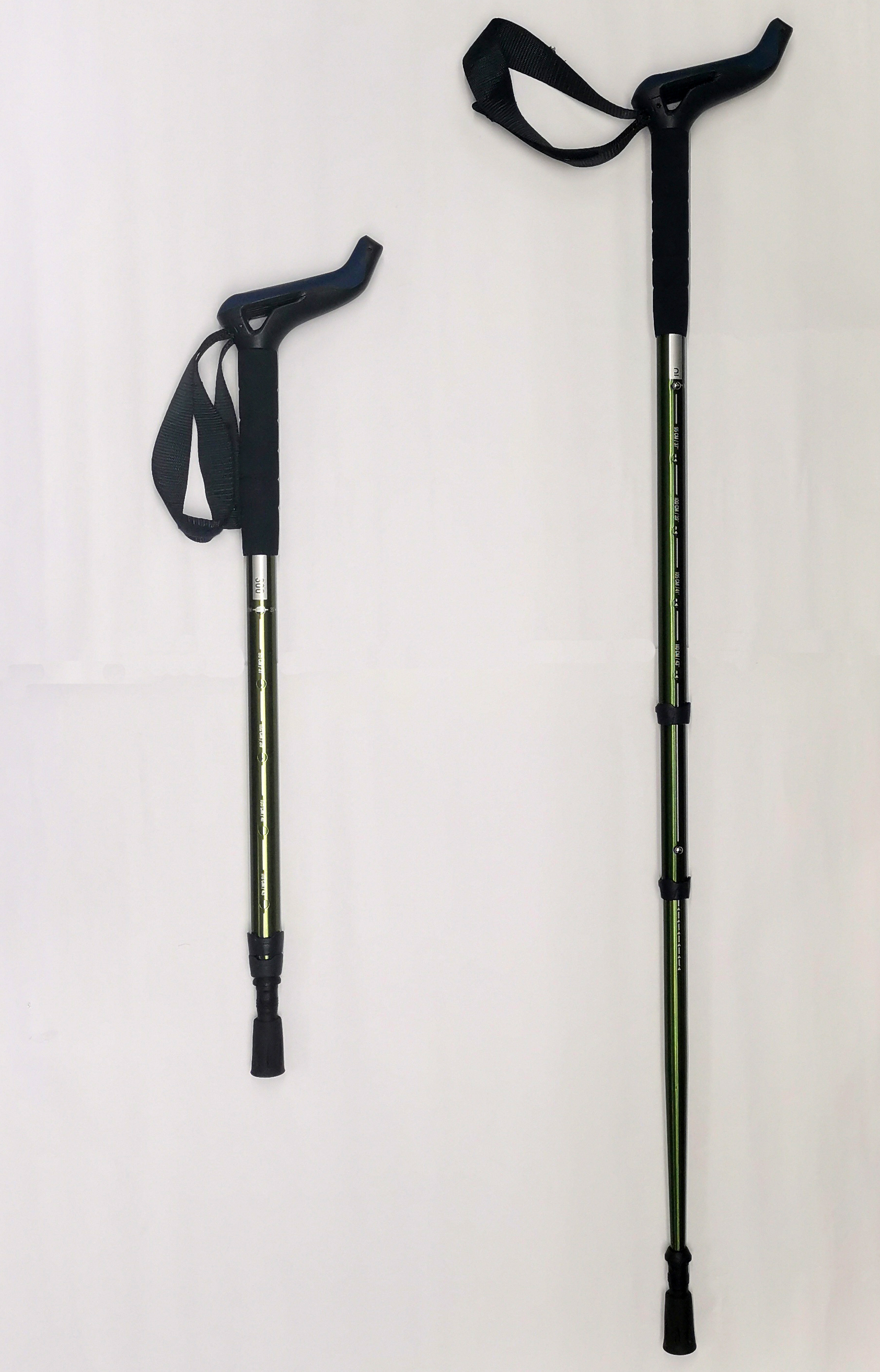

Supplement: S2 Fig — (TIF) [file pone.0303397.s002.tif]
