# Supplementary material for: Design and preliminary verification of a novel powered ankle–foot prosthesis: From the perspective of lower-limb biomechanics compared with ESAR foot
Source: PLoS One. 2024 Jun 7;19(6):e0303397. doi: 10.1371/journal.pone.0303397 (PMC11161064; doi:10.1371/journal.pone.0303397)
Supplement: S1 File — (DOCX) [file pone.0303397.s003.docx]

**Design and Preliminary Verification of a Novel Powered Ankle–Foot Prosthesis: From the Perspective of Lower-Limb Biomechanics Compared with ESAR Foot**

Jingjing Liu^1,2,*^, Jingang Liu^2^, Pei Yi Cheah^3^, Mouaz Al Kouzbary^1^, Hamza Al Kouzbary^1^, Selina X. Yao^4^, Hanie Nadia Shasmin^1^, Nooranida Arifin^1^, Nasrul Anuar Abd Razak^1^, and Noor Azuan Abu Osman^1,5,*^

^1^ Centre for Applied Biomechanics, Department of Biomedical Engineering, Faculty of Engineering, Universiti Malaya, 50603 Kuala Lumpur, Malaysia.

^2^ School of Mechanical Engineering and Mechanics, Xiangtan University, Xiangtan, Hunan 411105, China.

^3^ Centre for Sports and Exercise Science, Universiti Malaya, 50603 Kuala Lumpur, Malaysia.

^4^ Department of Mechanical Engineering, University of Vermont, Burlington, Vermont, 05405, USA.

^5^ The Chancellery, Universiti Tenaga Nasional, 43000 Kajang, Malaysia.

^*^ Correspondence Authors:

Email: [kva190023@siswa.um.edu.my](mailto:kva190023@siswa.um.edu.my) (Jingjing L.)

[azuan@um.edu.my](mailto:azuan@um.edu.my) (N.A.A.O.)

**Elastic Elements’ Optimisation Method**

The dynamic model of the unidirectional parallel elastic actuator with series elastic element is illustrated in Fig 1, and the dynamic equations of the SE+UPEA can be conducted according to the Newton–Euler Equations.


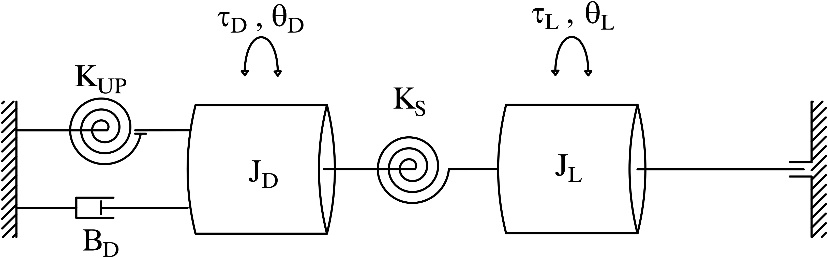


**Fig 1. Dynamic model of SE+UPEA.**

$$\begin{aligned} \text{J}_{\text{D}}{\ddot{\text{θ}}}_{\text{D}}\text{=}\text{τ}_{\text{D}}\text{+}\text{T}_{\text{B}}\text{+}\text{T}_{\text{S}}\text{+}\text{T}_{\text{UP}}\#\left( 1 \right) \end{aligned}$$

$$\begin{aligned} \text{J}_{\text{L}}{\ddot{\text{θ}}}_{\text{L}}\text{=}\text{τ}_{\text{L}}-\text{T}_{\text{S}}\#\left( 2 \right) \end{aligned}$$

where $\text{J}_{\text{D}}$ and $\text{J}_{\text{L}}$ are the rotational inertias or the equivalent ones of the driver and the load ends, respectively, $\text{θ}_{\text{D}}$ (${\dot{\text{θ}}}_{\text{D}}$ and ${\ddot{\text{θ}}}_{\text{D}}$) and $\text{θ}_{\text{L}}$ (${\dot{\text{θ}}}_{\text{L}}$ and ${\ddot{\text{θ}}}_{\text{L}}$) are the angular displacements (velocities and accelerations) of both ends, $\text{τ}_{\text{D}}$ is the torque on the driver end, i.e., the torque outputted by the transmission mechanism, $\text{τ}_{\text{L}}$ is the torque load on the load end, i.e. the torque load on the ankle joint during the operation of the prosthesis, $\text{T}_{\text{B}}$, $\text{T}_{\text{S}}$, and $\text{T}_{\text{UP}}$, which are the torques produced by the motor damping, series spring, and unidirectional parallel spring, respectively, are calculated based on Hooke’s Law.

$$\begin{aligned} \text{T}_{\text{B}}\text{=}-\text{B}_{\text{D}}{\dot{\text{θ}}}_{\text{D}}\#\left( 3 \right) \end{aligned}$$

$$\begin{aligned} \text{T}_{\text{S}}\text{=}-\text{K}_{\text{S}}\left( \text{θ}_{\text{D}}-\text{θ}_{\text{L}} \right)\#\left( 4 \right) \end{aligned}$$

$$\begin{aligned} \text{T}_{\text{UP}}\text{=}\left\{ \begin{aligned} \text{0} \\ -\text{K}_{\text{UP}}\left( \text{θ}_{\text{D}}-\text{θ}_{\text{UP}} \right) \end{aligned} \right.\text{ }\begin{aligned} \left( \text{θ}_{\text{D}}\text{≤}\text{θ}_{\text{UP}} \right) \\ \left( \text{θ}_{\text{D}}\text{>}\text{θ}_{\text{UP}} \right) \end{aligned}\#\left( 5 \right) \end{aligned}$$

where $\text{B}_{\text{D}}$ is the damping coefficient of the motor, $\text{K}_{\text{S}}$ is the stiffness of the series spring (SE), $\text{K}_{\text{UP}}$ and $\text{θ}_{\text{UP}}$ are the stiffness and equilibrium position of the unidirectional parallel spring (UPE), respectively.

In the ideal state, the powered ankle-foot prosthesis should be able to output the angular displacement of the ankle joint ($\text{θ}_{\text{L}}$) under the torque load ($\text{τ}_{\text{L}}$). Therefore, the driver end should have the capacity to output corresponding displacement (velocity) and torque, $\text{θ}_{\text{D}}$ (${\dot{\text{θ}}}_{\text{D}}$) and $\text{τ}_{\text{D}}$. First, the required output displacement can be solved by Eq. (2), which is shown in Eq. (6), and the output velocity and acceleration of the driver end (${\dot{\text{θ}}}_{\text{D}}$ and ${\ddot{\text{θ}}}_{\text{D}}$) can be gained by taking the first and second derivatives of both sides of Eq. (6). Next, all results are used to solve the required output torque, as shown in Eq. (7).

$$\begin{aligned} \text{θ}_{\text{D}}\text{=}\left( {-\text{τ}}_{\text{L}}\text{+}\text{J}_{\text{L}}{\ddot{\text{θ}}}_{\text{L}}\text{+}\text{K}_{\text{S}}\text{θ}_{\text{L}} \right)/{\text{K}_{\text{S}}}\#\left( 6 \right) \end{aligned}$$

$$\begin{aligned} \text{τ}_{\text{D}}\text{=}\text{J}_{\text{D}}{\ddot{\text{θ}}}_{\text{D}}\text{+}\text{B}_{\text{D}}{\dot{\text{θ}}}_{D}\text{+}\text{K}_{\text{S}}\left( \text{θ}_{\text{D}}-\text{θ}_{\text{L}} \right)-\text{T}_{\text{UP}}\#\left( 7 \right) \end{aligned}$$

According to Eqs. (6) and (7), the parameters of elastic elements will influence the required performance of the driver end under the specific ankle joint’s kinematics and kinetics. Thereinto, the required displacement (velocity) output by the driver end is only determined by the stiffness of the SE, but the parameters of both the SE and UPE influence the requirement of the output torque. Therefore, the stiffness of the SE is optimised to minimise the maximum of the absolute value of the velocity requirement, and then the stiffness and equilibrium position of the UPE is optimised to minimise the maximum of the absolute value of the torque requirement.

In this study, the optimisations of the elastic elements’ parameters are completed under the condition of walking on the level ground. The sources of the input parameters to the optimisations are listed as follows.

First, the inertia of the ankle joint in three stages of walking, which are measured and reported in Lee and Hogan [1], Rouse et al. [2], and Shorter and Rouse [3], respectively, is employed as the rotational inertia of the load end ($\text{J}_{\text{L}}$). The combined results are illustrated in Fig 2.


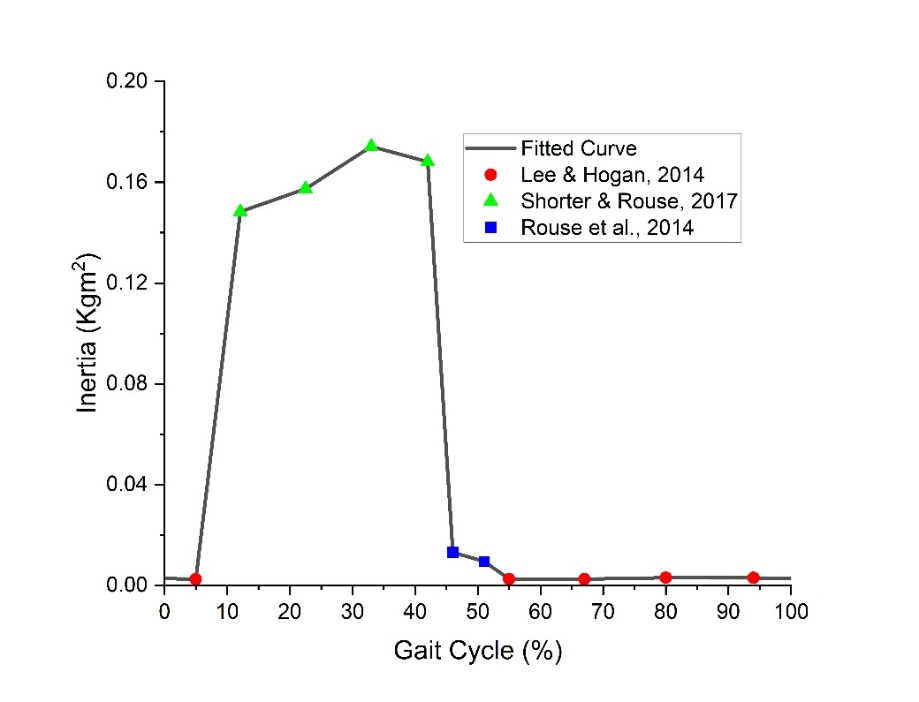


**Fig 2. Inertia of the ankle joint during normal walking on the level ground.**

Second, the angular displacement of the ankle joint ($\text{θ}_{\text{L}}$) is recorded from the first trial (normal walking) in this study. The results are in the format of average values plus/minus one standard deviation, as shown in Fig 3.


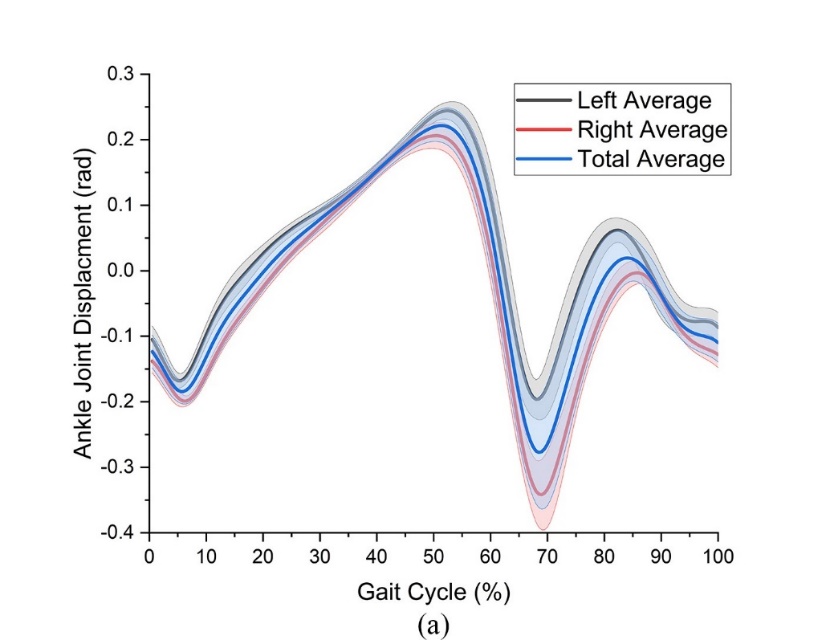


**Fig 3. Ankle joint displacement from the normal walking trial.**

Third, the torque load on the ankle joint ($\text{τ}_{\text{L}}$) is approximately calculated based the kinematic and kinetic data that are also recorded from the first trial in this study. An important assumption in this approximate method is that the foot is regarded as a rigid triangular segment. As shown in Fig 4(a), the length of the long side of the foot triangle is decided by the span of centre of pressure (COP) recorded by the force plate during the stance phase, and the static coordinates of the ankle joint $\left( X_{ankle},Y_{ankle} \right)$ are measured at the standing posture. As shown in Fig 4(b), at any moment in the stance phase, the coordinates of COP are $\left( X_{COP},0 \right)$, if the ground is $Z=0$. As the angle between the foot segment and the ground, which is termed foot pitch angle, being $\theta$, the torque load is calculated by Eq. (8). The dorsiflexion and plantarflexion have a positive and negative foot pitch angle, respectively.


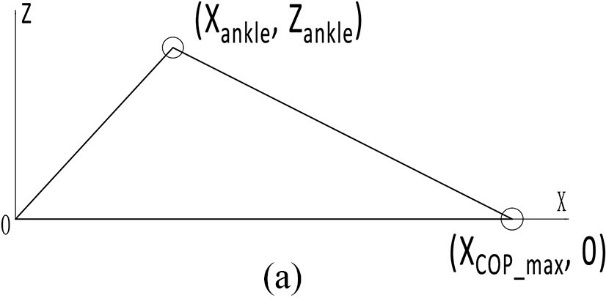

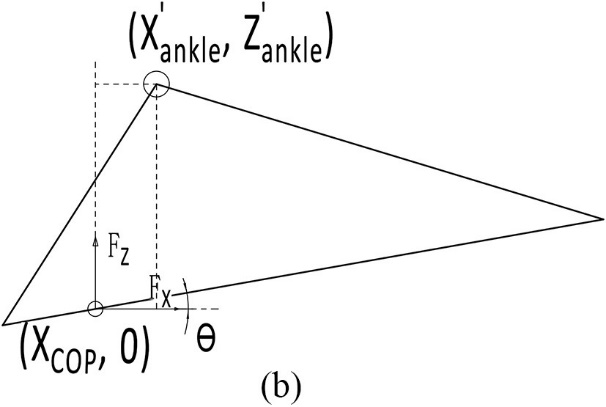


Fig 4. Diagram of the approximate method of calculating the torque load on the ankle joint in the sagittal plane.

$$\begin{aligned} \tau_{load}=F_{X}\left[ \left( X_{COP}-X_{ankle} \right)\sin\left( -\theta\right)+Z_{ankle}\cos\left( -\theta\right) \right] \\ -F_{Z}\left[ \left( X_{ankle}-X_{COP} \right)\cos\left( -\theta\right)+Z_{ankle}\sin\left( -\theta\right) \right] \#\left( 8 \right) \end{aligned}$$

where $F_{Z}$ and $F_{X}$ are the components of GRF along Z and X axis, respectively.

The COP along the X axis (COP_X_) and the components of GRF along the Z and X axes obtained from the trial of NW are illustrated in Figs 5-7, respectively. For ease of understanding, the total average COP_X_ is projected to the bottom side of the foot triangle in Fig 5. Both the error bar and shadow area reflect the average values plus/minus one standard deviation. The results are averaged based on the percentage of the stance phase. The positive and negative value of $F_{X}$ means the direction of the force is the same and opposite to the direction of walking, respectively. The calculated result of the torque load on the ankle joint is illustrated in Fig 8.

Finally, the parameters of the motor and transmission ($\text{J}_{\text{D}}$ and $\text{B}_{\text{D}}$) are obtained from the official datasheet and three-dimensional model. Based on all the above parameters, Eqs. (6) and (7) are modelled in Simulink (The MathWorks, Inc., U.S.A), and the abovementioned two-step optimisation is conducted by the application of Response Optimiser Toolbox to obtain the parameters of elastic elements.


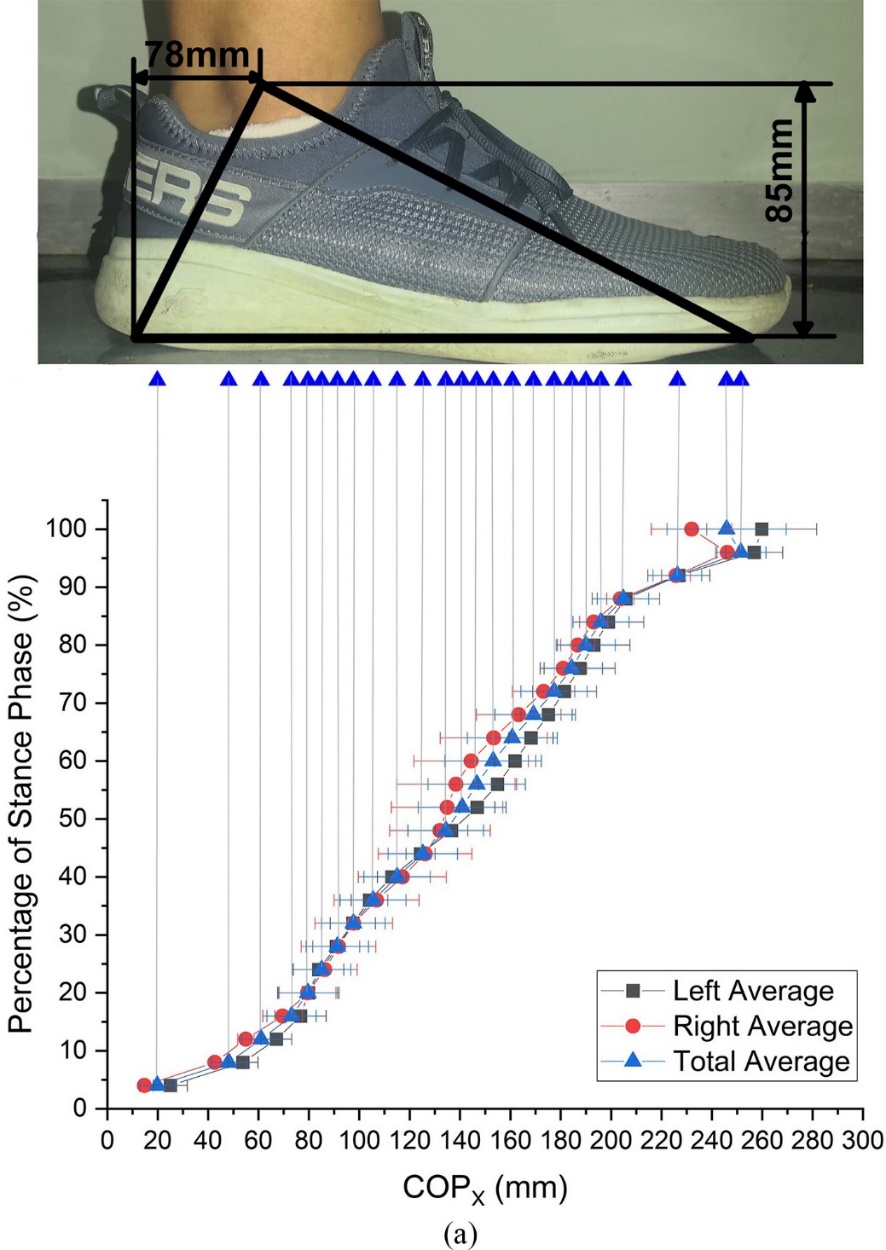


**Fig 5. COP from the normal walking trial.**


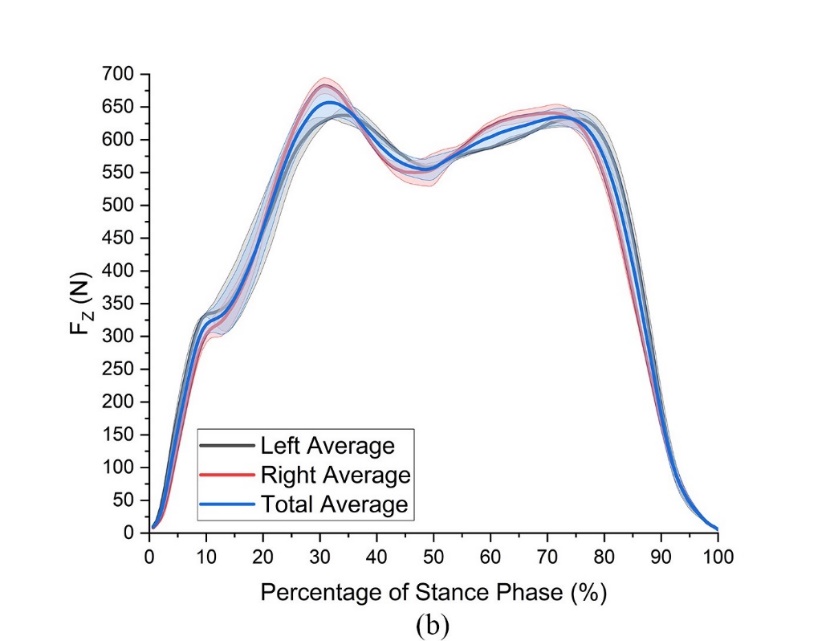


**Fig 6. Z-axis component of GRF from the normal walking trial.**


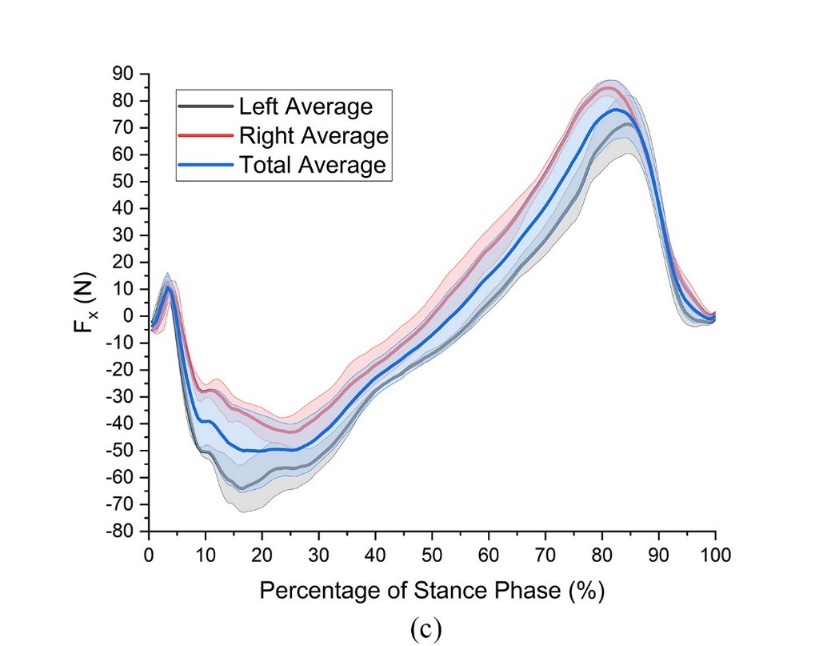


**Fig 7. X-axis component of GRF from the normal walking trial.**


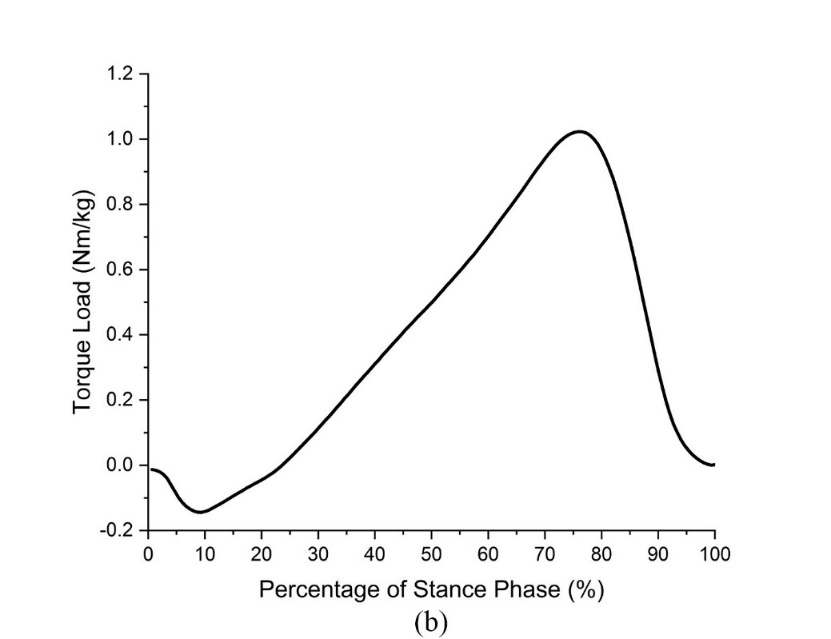


**Fig 8. Torque load on the ankle joint.**

**Reference**

1. Lee H, Hogan N. Time-varying ankle mechanical impedance during human locomotion. IEEE Transactions on Neural Systems and Rehabilitation Engineering. 2015;23(5):755-64. doi: 10.1109/TNSRE.2014.2346927.

2. Rouse EJ, Hargrove LJ, Perreault EJ, Kuiken TA. Estimation of human ankle impedance during the stance phase of walking. IEEE Transactions on Neural Systems and Rehabilitation Engineering. 2014;22(4):870-8. doi: 10.1109/TNSRE.2014.2307256.

3. Shorter AL, Rouse EJ. Mechanical impedance of the ankle during the terminal stance phase of walking. IEEE Transactions on Neural Systems and Rehabilitation Engineering. 2018;26(1):135-43. doi: 10.1109/TNSRE.2017.2758325.
